# Synthesis and PI3 Kinase Inhibition Activity of Some Novel Trisubstituted Morpholinopyrimidines

**DOI:** 10.3390/molecules23071675

**Published:** 2018-07-10

**Authors:** Emily W. Wright, Ronald A. Nelson, Yelena Karpova, George Kulik, Mark E. Welker

**Affiliations:** 1 Department of Chemistry, Wake Forest University, P.O. Box 7486, Winston-Salem, NC 27109, USA; wrighter11@gmail.com (E.W.W.); rnelson@wakehealth.edu (R.A.N.J.); 2 Department of Cancer Biology and Comprehensive Cancer Center, Wake Forest School of Medicine, Medical Center Blvd., Winston-Salem, NC 27157, USA; ykarpova@wakehealth.edu; 3 Life Sciences Program, College of Science, Alfaisal University, Riyadh 11533, Saudi Arabia

**Keywords:** triazine synthesis, PI3K inhibitor, prostate cancer

## Abstract

A number of new substituted morpholinopyrimidines were prepared utilizing sequential nucleophilic aromatic substitution and cross-coupling reactions. One of the disubstituted pyrimidines was converted into two trisubstituted compounds which were screened as PI3K inhibitors relative to the well-characterized PI3K inhibitor ZSTK474, and were found to be 1.5–3-times more potent. A leucine linker was attached to the most active inhibitor since it would remain on any peptide-containing prodrug after cleavage by prostate-specific antigen, and it did not prevent inhibition of AKT phosphorylation and hence the inhibition of PI3K by the modified inhibitor.

## 1. Introduction

Androgen-independent prostate cancer remains a major cause for prostate cancer-related deaths, since existing therapies provide only limited life extension [1,2,3,4,5,6,7]. Androgen ablation induces apoptosis in normal prostate epithelial cells, yet advanced androgen-independent cancer becomes resistant to apoptosis induced by androgen ablation and other cytotoxic therapies [8]. Numerous publications including papers from our group have demonstrated that several signaling pathways that operate downstream of receptor tyrosine kinases and G-protein-coupled receptors contribute to therapy resistance of prostate cancer [7,9,10,11,12,13,14,15]. Thus, activation of the PI3K/AKT (phosphoinositide 3-kinase/protein kinase B) pathway inhibits apoptosis in a wide range of cancers including prostate cancer. Inactivating mutations or deletions of the phosphatase and tensin homolog (PTEN) phosphatase that dephosphorylates PI3K products is the most common mechanism of constitutive activation of PI3K signaling that has been reported in 15–30% of primary and in the majority of metastatic prostate cancers [16,17,18,19,20,21,22].

Knocking out PTEN in the prostates of mice can trigger the development of prostate cancer [23]. Constitutive activation leads to “addiction” of cancer cells to the PI3K pathway; conversely, an inhibition of the PI3K pathway sensitizes prostate cancer cells to apoptosis [24,25,26]. Understanding PI3K–AKT–mTOR (mammalian target of rapamycin) signaling pathways and development of clinically useful inhibitors remains an active area of cancer research [27,28,29,30,31,32].

In 2012 [33] we reported that prodrugs containing PI3K inhibitors could be activated via peptide cleavage by prostate-specific antigen (PSA). Cheng and coworkers have also reported generation of prostate-selective PI3K inhibitors [34]. We have already shown specific inhibition of PI3K in PSA-secreting prostate cancer cells by a Mu-LEHSSKLQL-LY294002 (prodrug-LY294002) [33]. However, LY294002 inhibits PI3K at relatively high concentrations (25 µM) and is rapidly metabolized, so while its ease of synthesis made it a good choice for proof of concept, these pharmacological properties make it a poor candidate for in-vivo studies. 

We were confident that PSA would cleave the Mu-LEHSSKLQL peptide after glutamine (Q), therefore leaving leucine (L) attached to the PI3K inhibitor [35]. Computer modeling of interactions between PI3K inhibitors and the ATP-binding cleft of PI3K were used to identify positions at which the PSA substrate peptide could be attached so that linker plus leucine will not diminish PI3K inhibition. Following our review of PI3K inhibitor literature [36], we were most interested in preparing trisubstituted pyrimidines and triazines as the next test of this prodrug concept, and here we report our work on pyrimidines.

## 2. Results and Discussion

### 2.1. Chemistry

**Scheme 1:**
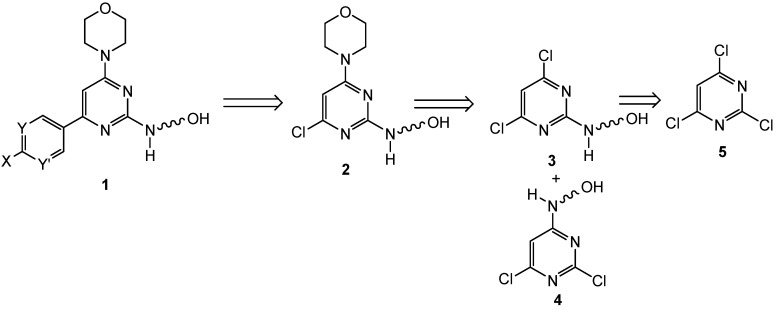
Retrosynthesis of trisubstituted pyrimidines.

Synthesis and characterization of trisubstituted triazines and pyrimidines remains an active area of PI3K inhibition research [37,38,39,40,41,42,43,44,45,46]. Since both core structures provide inhibitors that are quite active, we decided to prepare both trisubstituted triazines and trisubstituted pyrimidines, and here we report our work on the pyrimidines (Scheme 1). Target compounds reported here contain (1) a linker group terminating in a primary alcohol which can be used as a peptide linkage point; (2) morpholine (due to its known importance in PI3K inhibition [44]); and (3) a hydrogen-bonding aromatic or heteroaromatic group.

The triazine and pyrimidine cores offered simplicity in synthesis coupled with good PI3K inhibition activity [36,47,48,49,50,51,52,53,54,55,56,57,58]. Modelling as described previously [47,55,57] indicated that compounds containing the pyrimidine or triazine core substituted with a morpholine as well as an aromatic or heteroaromatic ring containing a hydrogen-bond donor exhibited Ki < 1 μM, good water solubility and hydrolytic stability. The third and final position on the pyrimidine/triazine core was left to be the attachment point for a functional group capable of serving as the link to the PSA-cleavable peptide sequence (Mu-LEHSSKLQL). Our initial work with LY 294002 analogs [33] used linkers that terminated in OH or NH_2_ groups so we initially chose those types of linkers again. In addition to this linker we were also interested in having a functional group in one of the other two core substituents that contained a heteroaromatic ring with a hydrogen-bonding substituent.

**Scheme 2:**
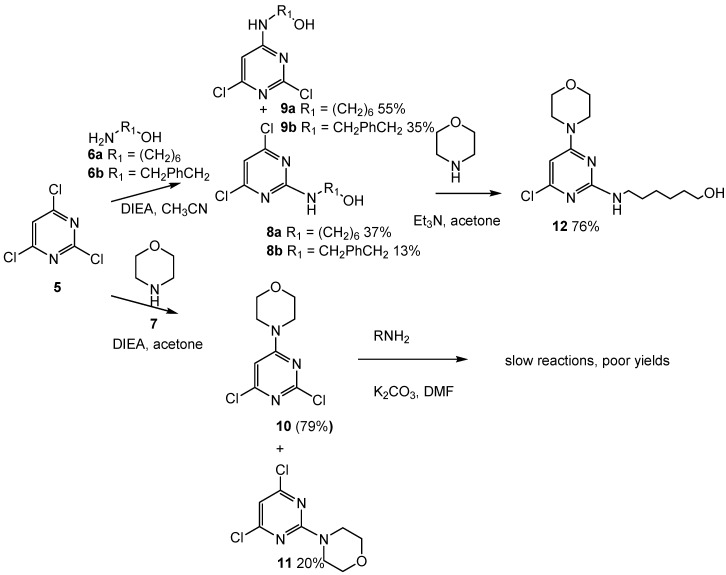
Nucleophilic aromatic substitution reactions of chloropyrimidines.

Given that we wanted to prepare trisubstituted pyrimidines containing a linker, an aromatic hydrogen-bond donor and a morpholine, we could conceivably initiate synthetic work by putting on any one of these three groups in step one. In practice, we envisioned replacing the last halogen on the pyrimidine via cross-coupling chemistry rather than nucleophilic substitution, so we left this to our third step. This decision meant we could initiate syntheses that started by adding linkers first or morpholine (Scheme 2) first.

### 2.2. First Addition Reaction (Scheme 2)

Sequential nucleophilic aromatic substitution reactions on 2,4,6-trichloropyrimidine present some challenges which are not present for the triazine core. Statistically, if one assumes all three chlorine-containing carbons are equally reactive, then the best one can hope for off the first nucleophilic addition is a 2:1 mixture of products. Separation can be effected then or sometimes others have then added a second nucleophile and separated the four products produced [46,59]. A second consideration is rate of reaction and isolated yields when one has the option of adding a primary amine or secondary amine as the first or second step in the sequence. In our hands, morpholine (**7**) added to trichloropyrimidine (**5**) much faster than the primary amines, and if we added morpholine (**7**) first then reactions of primary amines on a morpholine-substituted dichloropyrimidine (**10** or **11**) were very sluggish and hard to drive to completion. Given that, we decided to pursue adding primary amino alcohols to trichloropyrimidine first. We performed reactions using both 6-amino-1-hexanol (**6a**) and 4-aminomethylphenylmethanol (**6b**) and isolated much higher yields of products (**8a**/**9a**) from 6-amino-1-hexanol (**6a**) (Scheme 2). We decided to take the minor isomer (**8a**) on for convenience since subsequent nucleophilic additions (in our case here morpholine) to that compound can yield only one regioisomer (**12**) (whereas additions to **9a** or **10** would be expected to yield two regioisomers). We isolated **12** in excellent yield and avoided the careful chromatographic separations needed for **8**–**11** at this disubstituted pyrimidine stage.

### 2.3. Third Addition Reaction (Scheme 3)

**Scheme 3:**
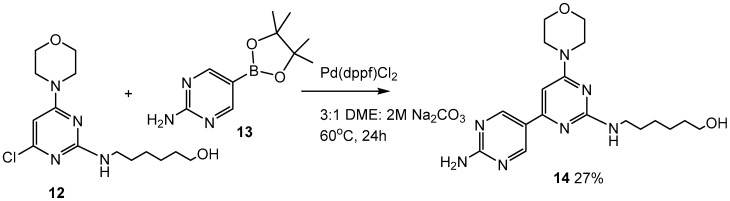
Cross-coupling reaction of disubstituted pyrimidine (**12**).

A variety of cross-coupling conditions were investigated in order to add the last heteroaromatic hydrogen-bonding substituent. For this work, we chose 2-aminopyrimidine-5-boronic acid pinacol ester (**13**) (Scheme 3). Pd(PPh_3_)_4_ (10 mol %) was used initially as a catalyst in DMSO, THF and DME (3:1 organic solvent: 2 M Na_2_CO_3_). DME proved superior and we observed no product when the 2 M Na_2_CO_3_ was eliminated. Pd(dppf)Cl_2_, Pd(OAc)_2_/PPh_3_, Pd(OAc)_2_/CuCl_2_/S-Phos and Pd(OAc)_2_/CuCl_2_/K_3_PO_4_/S-Phos were all investigated as catalysts and Pd(dppf)Cl_2_ provided the highest yield of cross-coupled product (**14**). Compound **14** proved to be as active a PI3K inhibitor as ZSTK 474 (see screening discussion below), and therefore we undertook preparation of its isomer as well as its preparation where leucine was added to the primary OH in the linker group. 

### 2.4. Attempt to Prepare an Isomer of ***14***

**Scheme 4:**
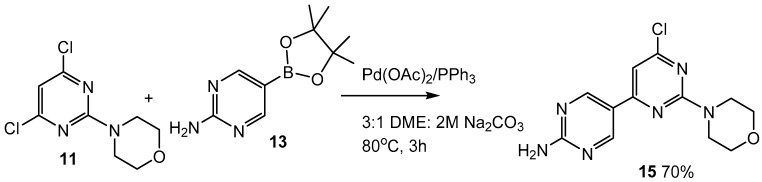
Cross-coupling of dichloromorpholinopyrimidine (**11**).

We attempted to prepare an isomer of the active compound (**14**) by first performing a cross-coupling reaction on morpholine-substituted pyrimidine (**11**), and this reaction proceeded in good yield to produce **15** (Scheme 4). However, attempts to add primary amines to **15**, with heat, Pd catalysts or Pd catalysts and heat proved unsuccessful.

### 2.5. Attempts to Prepare an Analog of ***14*** with a Terminal Alkyne Rather than Primary OH as Peptide Link Point

**Scheme 5:**
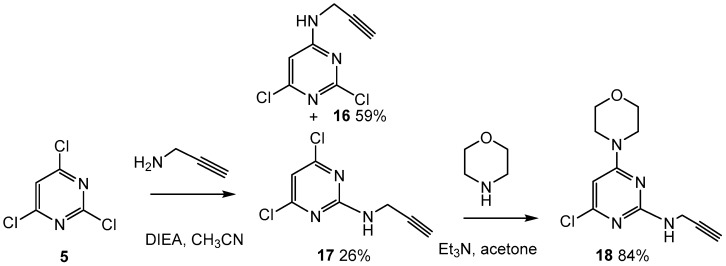
Use of propargyl amine as a potential peptide link point.

In our earlier work we had used a primary alcohol as a peptide attachment point when making PI3K-inhibitor prodrugs [33]. This necessitates an ester functional group for the attachment so we wanted to investigate other alternatives. We hoped that if we could prepare an analog of **14** with a terminal alkyne linker group then click chemistry would become an option for attaching peptide sequences. To test this option, we first added propargyl amine to trichloropyrimidine (**5**) as we had done for aminohexanol and this produced two isomers (**16** and **17**) as expected (Scheme 5). The symmetrical isomer (**17**) was taken on for the reasons outlined above and the morpholine-containing disubstituted pyrimidine (**18**) was isolated in high yield. Unfortunately, a variety of cross-coupling conditions for attachment of **13** to **18** (identical to the battery we had tried for **14**) produced none of the desired cross-coupled trisubstituted product. We also tried back-tracking here and attempted to cross-couple **13** to **17** under a variety of conditions but those also failed, leaving us to conclude that the terminal alkyne was not compatible with these conditions. 

### 2.6. Addition of Leucine to the Lead Compound (***14***)

**Scheme 6:**
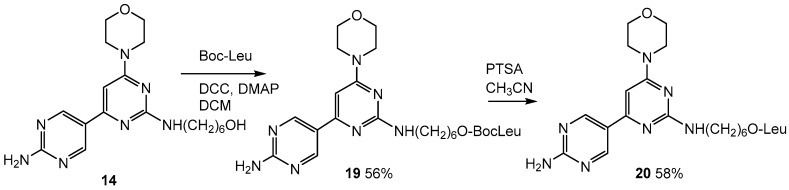
Addition of leucine to lead compound **14**.

Since compound **14** was quite active as a PI3K inhibitor (see screening discussion below), we undertook its preparation where leucine was added to the primary OH in the linker group. In our earlier work [33], we demonstrated that peptide (Mu-LEHSSKLQ) in prostate-specific prodrugs is cleaved between L and Q, so the leucine residue remains attached to the PI3K inhibitor. To mimic the prodrug cleaved by PSA, Boc-protected leucine was coupled to **14** to produce **19** in good yield and then the protecting group was removed to yield **20** (Scheme 6).

### 2.7. Biological Activity

The biological activities of new PI3K-inhibitor compounds (**14**) and (**20**) were tested in C4-2 prostate cancer cells with constitutive activation of PI3K/AKT pathway. C4-2 cells were derived by passaging of prostate cancer metastases-derived LNCaP cells through nude mice and characterized by increased propensity to form metastases in mice. The PI3K/AKT pathway is activated in these cells due to frame-shift mutation in the PTEN tumor-suppressor gene. Activation of PI3K leads to accumulation of phosphatidylinositol (3,4,5)-trisphosphate (PIP_3_) in the plasma membrane that recruits protein kinases AKT and PDK1 through binding to their PH domains. Interaction of PIP_3_ with the PH domain changes AKT conformation and opens access for PDK1 to phosphorylate at T308, and also allows phosphorylation at S473 by TORC2 complex. Thus, phosphorylation of AKT at S473 and T308 is routinely used to monitor the PI3K activity because it depends on activation of the PI3K pathway in most cells [59].

**Figure 1:**
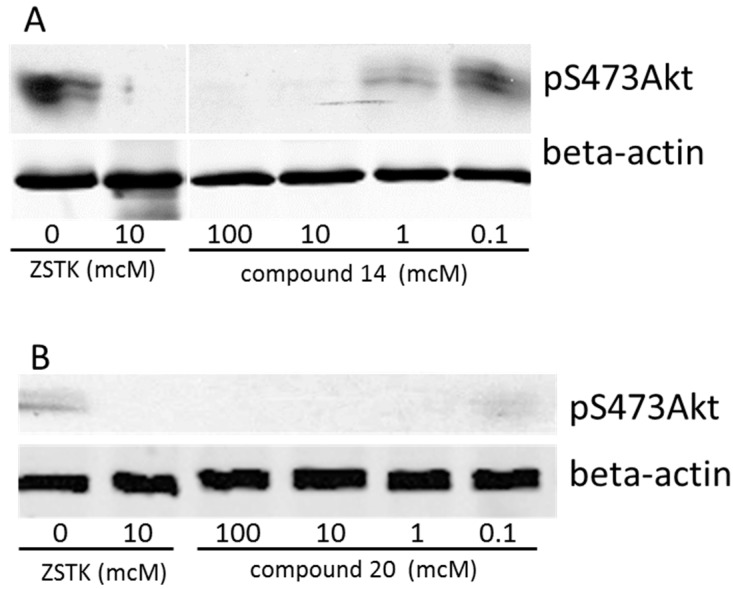
Representative Western blots of S473AKT phosphorylation in C4-2 prostate cancer cells. (**A**) C4-2 cells were treated with ZSTK474 (10 µM) or compound **14** (100, 10, 1, 0.1 µM); (**B**) C4-2 cells were treated with ZSTK474 (10 µM) or compound **20** (100, 10, 1, 0.1 µM). Equal loading is controlled by probing Western blot for beta-actin.

Phosphorylation of AKT at S473 in C4-2 cells has been used in this study to assess the PI3K inhibition. Figure 1 shows representative Western blots that illustrate inhibition of S473AKT phosphorylation by **14** and by **20**, which contains the leucine linker. Quantitative comparison of Western blots showed that IC_50_ values for **14** and **20** were 3.2- and 1.5-fold lower compared to ZSTK474, a widely used PI3K inhibitor.

## 3. Materials and Methods

### 3.1. Compound Synthesis

#### 3.1.1. General Methods

The general experimental methods used here were essentially the same as those we have described previously [33]. Copies of spectra that the data presented below are taken from are also included as supplementary materials.

#### 3.1.2. 6-((4,6-Dichloropyrimidin-2-yl)amino)hexan-1-ol (**8a**) and 6-((2,6-dichloropyrimidin-4-yl)amino)hexan-1-ol (**9a**)

Trichloropyrimidine (**5**) (0.917 g, 5.00 mmol) was dissolved in acetonitrile (20 mL). 6-Amino-1-hexanol (**6a**) (1.05 eq., 0.615 g, 5.25 mmol) and *N*,*N*-diisopropylethylamine (4 eq., 2.59 g, 0.020 mol) were added dropwise at 0 °C. The reaction mixture was allowed to warm to room temperature and stirred for three hours. The product was condensed by rotary evaporation and purified on silica using ethyl acetate and hexanes (1:1) to give 6-((4,6-dichloropyrimidin-2-yl)amino)hexan-1-ol (**8a**) (0.493 g, 1.87 mmol, 37%): R_f_ 0.45 (50% EtOAc in hexanes) and 6-((2,6-dichloropyrimidin-4-yl)amino)hexan-1-ol (**9a**) (0.730 g, 2.78 mmol, 55%): R_f_ 0.6 (50% EtOAc in hexanes) as clear oils. 6-((4,6-dichloropyrimidin-2-yl)amino)hexan-1-ol (**8a**): ^1^H-NMR (300 MHz, CDCl_3_) δ 6.58 (s, 1H), 5.43 (br s, 1H), 3.65 (t, *J* = 6 Hz, 2H), 3.42 (app q, *J* = 6 Hz, 2H), 1.59 (m, 6H), 1.40 (m, 2H), 1.34 (br s, 1H). ^13^C-NMR (75 MHz, CDCl_3_) (We should note that we rarely see the C bonded to 3 nitrogens due to ^14^N quadrupolar broadening) δ 162.14, 108.62, 62.71, 41.46, 36.68, 32.58, 29.16, 26.51, 25.38. Elem. anal. calcd. for C_10_H_15_N_3_OCl_2_: C, 45.47; H, 5.72; found: C, 45.72; H, 5.72. 6-((2,6-dichloropyrimidin-4-yl)amino)hexan-1-ol (**9a**): ^1^H-NMR (300 MHz, CDCl_3_) δ 6.26 (s, 1H), 5.60 (br s, 1H), 3.66 (t, *J* = 6 Hz, 2H), 3.26 (br s, 2H), 1.61 (m, 4H), 1.66 (br s, 1H), 1.42 (m, 4H). ^13^C-NMR (75.47 MHz, CDCl_3_) δ 164.20, 160.86, 159.67, 62.67, 41.95, 32.43, 28.85, 26.50, 25.36. HRMS [M + H]^+^ calcd. for C_10_H_15_N_3_OCl_2_: 264.0665; found: 264.0665.

#### 3.1.3. (4-(((2,6-Dichloropyrimidin-4-yl)amino)methyl)phenyl)methanol, (**8b**) (4-(((4,6-dichloropyrimidin-2-yl)amino)methyl)phenyl)methanol, (**9b**)

2,4,6-Trichloropyrimidine (**5**) (0.158 g, 0.86 mmol) was dissolved in MeCN (3 mL) and cooled to 0 °C. 4-(Aminomethyl)phenyl)methanol (**6b**) (1.1 eq., 0.130 g, 0.95 mmol) and DIEA (4 eq., 0.445 g, 3.44 mmol) were added before warming the reaction to room temperature while stirring vigorously for 15 min. The reaction was concentrated via rotary evaporation and high vacuum. Two products were purified via column chromatography (50% ethyl acetate in hexanes) to yield major isomer (**9b**) (0.086 g, 0.30 mmol, 35%) and minor isomer (**8b**) (0.032 g, 0.112 mmol, 13%). Data for **9b**: Elem. anal. for C_12_H_11_Cl_2_N_3_O: C, 50.72 (found 50.72); H, 3.90 (4.11). ^1^H-NMR (300 MHz, DMSO-*d*_6_) δ 8.86 (s, 0H), 8.62 (s, 1H), 7.34–7.18 (m, 4H), 6.58 (s, 1H), 5.14 (t, *J* = 5.7 Hz, 1H), 4.49 (dd, *J* = 8.5, 5.7 Hz, 4H). ^13^C-NMR (75 MHz, DMSO-*d*_6_) δ 163.98, 159.03, 156.92, 141.55, 136.23, 127.29, 126.77, 126.54, 102.71, 62.59, 43.67, 40.21. HRMS [M]^+^ calculated for C_12_H_11_N_3_OCl_2_, 284.0279; found 284.0366. Data for **8b**: Elem. anal. calcd. for C_12_H_11_Cl_2_N_3_O: C, 50.72 (found 51.41); H, 3.90 (4.17). ^1^H-NMR (300 MHz, DMSO-*d*_6_) δ 8.65 (t, *J* = 6.3 Hz, 1H), 7.25 (s, 24H), 6.88 (s, 1H), 5.11 (td, *J* = 5.7, 0.9 Hz, 1H), 4.46 (dd, *J* = 6.0, 4.4 Hz, 4H), 3.27 (d, *J* = 7.2 Hz, 0H). ^13^C-NMR (75 MHz, DMSO-*d*_6_) δ 161.46, 161.03, 160.88, 141.15, 137.05, 126.88, 126.44, 107.71, 62.65, 43.92, 39.37, 39.05, 38.80. HRMS [M]^+^ calcd. for C_12_H_11_N_3_OCl_2_, 284.0279; found 284.0359.

#### 3.1.4. 4-(2,6-Dichloropyrimidin-4-yl)morpholine, (**10**) and 4-(4,6-dichloropyrimidin-2-yl)morpholine, (**11**)

2,4,6-Trichloropyrimidine (**5**) (3.158 g, 17.22 mmol) and acetone (60 mL) were combined and cooled to 0 °C. Morpholine (**7**) (1.05 eq., 1.576 g, 18.09 mmol) was added and the solution stirred at 0 °C for 15 min then warmed to room temperature for another 15 min. The reaction was monitored by TLC analysis (20% ethyl acetate in hexanes). The reaction was then concentrated via rotary evaporation and further dried under high vacuum. Product was purified using silica column chromatography with 20% ethyl acetate in hexanes as the eluent. **10** (major regioisomer) (3.183 g, 13.6 mmol, 79%). Elem. anal. for C_8_H_9_N_3_OCl_2_: C, 41.05 (found 42.27); H, 3.88 (found 4.06). ^1^H-NMR (300 MHz, CDCl_3_) δ 6.40 (s, 1H), 3.82–3.70 (m, 4H), 3.65 (m, 4H). HRMS [M]^+^ calcd. for C_8_H_9_N_3_OCl_2_, 234.0201; found 234.0196. **11** (minor regioisomer) (0.806 g, 3.444 mmol, 20%). ^1^H-NMR (300 MHz, chloroform-*d*) δ 6.56 (s, 1H), 3.85–3.60 (m, 8H). ^13^C-NMR (101 MHz, chloroform-*d*) δ 161.75, 160.55, 108.31, 66.59, 44.39. HRMS [M]^+^ calcd. for C_8_H_9_N_3_OCl_2_, 234.0201; found 234.0196.

#### 3.1.5. 6-((4-Chloro-6-morpholinopyrimidin-2-yl)amino)hexan-1-ol (**12**)

6-((4,6-dichloropyrimidin-2-yl)amino)hexan-1-ol (**8a**) (0.200 g, 0.757 mmol) was dissolved in acetone (20 mL). Morpholine (**7**) (1.05 eq., 0.069 g, 0.795 mmol) and triethylamine (4 eq., 0.306 mL, 3.03 mmol) were added and the solution was stirred overnight at room temperature (24 h). The product was condensed by rotary evaporation and purified on silica using ethyl acetate to give 6-((4-chloro-6-morpholinopyrimidin-2-yl)amino)hexan-1-ol (**12**) (0.181 g, 0.576 mmol, 76%): R_f_ 0.7 (100% EtOAc) as a clear oil. ^1^H-NMR (300 MHz, CDCl_3_) δ 5.84 (s, 1H), 5.22 (br s, 1H), 3.74 (m, 4H), 3.62 (t, *J* = 7 Hz, 2H), 3.55 (m, 4H), 3.33 (q, *J* = 6 Hz, 2H), 2.27 (br s, 1H), 1.57 (m, 4H), 1.37 (m, 4H). ^13^C-NMR (75 MHz, CDCl_3_) δ 163.66, 161.60, 160.28, 90.97, 66.48, 62.74, 44.33, 41.25, 32.63, 29.52, 26.69, 25.50. Elem. anal. calcd. for C_14_H_23_N_4_O_2_Cl: C, 53.41%; H, 7.36%; found: C, 53.50%; H, 7.29%.

#### 3.1.6. 6-((2′-Amino-6-morpholino-[4,5′-bipyrimidin]-2-yl)amino)hexan-1-ol (**14**)

6-((4-chloro-6-morpholinopyrimidin-2-yl)amino)hexan-1-ol (**12**) (0.150 g, 0.477 mmol) was dissolved in 3:1 DME/2 M Na_2_CO_3_ (8 mL) in a sealed tube. Nitrogen was bubbled through the solution for two minutes. 2-Aminopyrimidine-5-boronic acid pinacol ester (**13**) (2 eq., 0.208 g, 0.955 mmol) and (1,1′-bis(diphenylphosphino)ferrocene)palladium(II) dichloride (0.15 eq., 0.058 g, 0.072 mmol) were added and nitrogen was bubbled through the solution again for five minutes. The tube was sealed and stirred in an oil bath at 60 °C for 24 h. The mixture was allowed to cool to room temperature and quenched with aqueous sodium carbonate. The solution was extracted with ethyl acetate (2 × 10mL), dried with anhydrous sodium sulfate, and condensed by rotary evaporation. The product was purified on silica using 5% ethanol in ethyl acetate to produce 6-((2′-amino-6-morpholino-[4,5′-bipyrimidin]-2-yl)amino)hexan-1-ol (**14**) (0.048 g, 0.129 mmol, 27%): R_f_ 0.15 (5% EtOH in EtOAc) as a light brown powder. ^1^H-NMR (300 MHz, CDCl_3_) δ 8.84 (s, 2H), 6.11 (s, 1H), 5.50 (s, 2H), 5.18 (br s, 1H), 3.79 (m, 4H), 3.64 (m, 6H), 3.42 (q, *J* = 6 Hz, 2H), 2.62 (br s, 1H), 1.61 (m, 4H), 1.45 (m, 4H). ^13^C-NMR (75 MHz, CDCl_3_) δ 163.74, 163.31, 162.29, 159.93, 157.12, 122.42, 87.13, 66.63, 62.75, 44.33, 41.30, 32.68, 29.72, 26.78, 25.53. HRMS [M + H]^+^ calcd for C_18_H_27_N_7_O_2_: 374.2300; found: 374.2300.

#### 3.1.7. 6-Chloro-2-morpholino-[4,5′-bipyrimidin]-2′-amine (**15**)

4-(4,6-Dichloropyrimidin-2-yl)morpholine (**11**) (0.100 g, 0.429 mmol) and 2-aminopyrimidine-5-boronic acid pinacol ester (**13**) (1.8 eq., 0.170 g, 0.769 mmol) were dissolved in 3:1 DME/2 M Na_2_CO_3_ (8 mL). This solution was degassed for two minutes with nitrogen. Palladium acetate (0.15 eq., 0.014 g, 0.062 mmol) and triphenylphosphine (0.15 eq., 0.105 g, 0.064 mmol) were added and nitrogen was bubbled through the solution for 5 min. The reaction mixture was refluxed at 80 °C for three hours. It was then allowed to cool to room temperature and quenched with aqueous sodium carbonate. The solution was extracted with ethyl acetate (2 × 15 mL), dried with anhydrous sodium sulfate, and condensed by rotary evaporation. A 1000 μM prep-TLC plate was used to purify the product using ethyl acetate as the mobile phase to give 6-chloro-2-morpholino-[4,5′-bipyrimidin]-2′-amine (**15**) (0.088 g, 0.300 mmol, 70%): R_f_ 0.3 (EtOAc) as a light-brown powder. ^1^H-NMR (300 MHz, CDCl_3_) δ 8.91 (s, 2H), 6.83 (s, 1H), 5.43 (s, 2H), 3.88 (m, 4H), 3.78 (m, 4H). ^13^C-NMR (75 MHz, CDCl_3_) δ 163.81, 161.74, 157.63, 132.04, 128.48, 120.53, 103.20, 66.74, 44.34.

#### 3.1.8. 2-(But-3-yn-1-yl)-4,6-dichloropyrimidine (**17**) and 4-(but-3-yn-1-yl)-2,6-dichloropyrimidine (**16**)

Trichloropyrimidine (**5**) (0.917 g, 5.00 mmol) was dissolved in acetonitrile (20 mL). Propargylamine (1.05 eq., 0.547 g, 5.25 mmol) and *N*,*N*-diisopropylethylamine (4 eq., 2.59 g, 0.020 mol) were added dropwise at 0 °C. The reaction mixture was allowed to warm to room temperature and stirred for three hours. The product was condensed by rotary evaporation and purified on silica using ethyl acetate and hexanes (1:5) to give 2-(but-3-yn-1-yl)-4,6-dichloropyrimidine (**17**) (0.261 g, 1.29 mmol, 26%): R_f_ 0.5 (1:5 EtOAc in hexanes) and 4-(but-3-yn-1-yl)-2,6-dichloropyrimidine (**16**) (0.595 g, 2.95 mmol, 59%): R_f_ 0.4 (1:5 EtOAc in hexanes) as light-yellow powders. 2-(But-3-yn-1-yl)-4,6-dichloropyrimidine (**17**): ^1^H-NMR (300 MHz, CDCl_3_) δ 6.61 (s, 1H), 5.44 (br s, 1H), 4.16 (dd, *J* = 6, 3 Hz, 2H), 2.19 (t, *J* = 3 Hz, 1H). ^13^C-NMR (75 MHz, CDCl_3_) δ 162.30, 160.89, 110.10, 79.21, 71.71, 31.47. HRMS [M + H]^+^ calcd for C_7_H_5_N_3_Cl_2_: 201.9930; found: 201.9930. 4-(But-3-yn-1-yl)-2,6-dichloropyrimidine (**16**): ^1^H-NMR (300 MHz, CDCl_3_) δ 6.31 (s, 1H), 5.33 (br s, 1H), 4.09 (br s, 2H), 2.25 (t, *J* = 3 Hz, 1H). ^13^C-NMR (75 MHz, CDCl_3_) δ 171.25, 163.75, 159.87, 101.27, 72.83, 60.43, 31.44. HRMS [M + H]^+^ calcd. for C_7_H_5_N_3_Cl_2_: 201.9930; found: 201.9932.

#### 3.1.9. 4-(2-(But-3-yn-1-yl)-6-chloropyrimidin-4-yl)morpholine (**18**)

2-(But-3-yn-1-yl)-4,6-dichloropyrimidine (**4**) (0.231 g, 1.64 mmol) was dissolved in acetone (20 mL). Morpholine (1.05 eq., 0.141 g, 1.04 mmol) and triethylamine (4 eq., 0.306 mL, 3.03 mmol) were added and the solution was stirred overnight at room temperature (24 h). A pure product precipitated out with the addition of cold distilled water (60 mL) which was filtered and dried by vacuum to give 4-(2-(but-3-yn-1-yl)-6-chloropyrimidin-4-yl)morpholine (**6**) (0.345 g, 1.64 mmol, 84%): R_f_ 0.5 (50% EtOAc in hexanes) as a white powder. ^1^H-NMR (300 MHz, CDCl_3_) δ 5.92 (s, 1H), 5.12 (br s, 1H), 4.16 (dd, *J* = 5, 2 Hz, 2H), 3.75 (m, 4H), 3.58 (t, *J* = 5 Hz, 4H), 2.19 (m, 1H). ^13^C-NMR (75 MHz, CDCl_3_) δ 162.95, 160.99, 159.88, 92.50, 79.67, 71.62, 66.79, 44.25, 30.90. HRMS [M + H]^+^ calcd. for C_11_H_13_N_4_OCl: 253.0850; found: 253.0849.

#### 3.1.10. 6-((2′-Amino-6-morpholino-[4,5′-bipyrimidin]-2-yl)amino)hexyl (tert-butoxycarbonyl)-l-leucinate (**19**)

6-((2′-Amino-6-morpholino-[4,5′-bipyrimidin]-2-yl)amino)hexan-1-ol (**14**) (0.050 g, 0.134 mmol) was dissolved in dichloromethane (DCM), (15 mL). *N*-α-tert-Butoxycarbonyl-l-leucine (2 eq., 0.062 g, 0.268 mmol), *N*,*N*′-dicyclohexylcarbodiimide (2 eq., 0.055 g, 0.268 mmol), and dimethylaminopyridine (1 eq., 0.016 g, 0.134 mmol) were added and the mixture was stirred at room temperature for 20 h under nitrogen. The reaction was diluted with DCM (30 mL) and extracted with three separate aqueous solutions: Cold 1 M HCL (30 mL), saturated NaHCO_3_ (30 mL) and brine (30 mL). The organic fraction was dried with sodium sulfate and condensed by rotary evaporation. The product was purified on silica using 5% ethanol in ethyl acetate to yield 6-((2′-amino-6-morpholino-[4,5′-bipyrimidin]-2-yl)amino)hexyl (tert-butoxycarbonyl)-l-leucinate (**19**) (0.044 g, 0.075 mmol, 56%): R_f_ 0.5 (5% EtOH in EtOAc) as an off-white powder. ^1^H-NMR (300 MHz, CDCl_3_) δ 8.85 (s, 2H), 6.12 (s, 1H), 5.50 (br s, 2H), 5.13 (br s, 1H), 5.00 (br s, 1H), 4.31 (br s, 1H), 4.13 (m, 2H), 3.79 (m, 4H), 3.62 (m, 4H), 3.43 (q, *J* = 4, 2H), 1.67-1.45 (m, 11H), 1.44 (s, 9H), 0.93 (m, 6H). ^13^C-NMR (75 MHz, CDCl_3_) δ 173.61, 163.80, 163.42, 162.39, 160.14, 157.10, 155.42, 122.51, 87.08, 79.72, 66.62, 65.11, 52.16, 44.33, 41.87, 41.26, 29.76, 28.33, 26.61, 25.65, 24.80, 22.80, 21.96. HRMS [M]^+^ calcd. for C_28_H_46_N_8_O_5_: 587.3665; found: 587.3665.

#### 3.1.11. 6-((2′-Amino-6-morpholino-[4,5′-bipyrimidin]-2-yl)amino)hexyl-l-leucinate (**20**)

6-((2′-Amino-6-morpholino-[4,5′-bipyrimidin]-2-yl)amino)hexyl (tert-butoxycarbonyl)-l-leucinate (**19**) (0.130 g, 0.222 mmol) was dissolved in acetonitrile (20 mL). *p*-Toluenesulfonic acid monohydrate (2 eq., 0.084 g, 0.444 mmol) was added and the solution was stirred under nitrogen at room temperature for 48 h. *p*-Toluenesulfonic acid monohydrate (1 eq., 0.042 g, 0.222 mmol) was added and the solution continued to stir for 24 h. At this point the reaction was monitored by LC/MS showing approximately 66% product creation. Additional *p*-toluenesulfonic acid monohydrate (3 eq., 0.126 g, 0.666 mmol) was added and the reaction was stirred for an additional 48 h. The reaction mixture was condensed by rotary evaporation. The solid residue was then added to cold water (10 mL) and extracted with ethyl acetate (2 × 10 mL). To the aqueous fraction, saturated sodium carbonate (20 mL) was added and this was extracted with three portions of ethyl acetate (20 mL). The organic fractions were combined and condensed by rotary evaporation and high vacuum to give 6-((2′-amino-6-morpholino-[4,5′-bipyrimidin]-2-yl)amino)hexyl-l-leucinate (**20**) (0.062 g, 0.127 mmol, 58%): R_f_ 0.4 (5% EtOH in EtOAc) as a clear oil. ^1^H-NMR (300 MHz, CDCl_3_) δ 8.85 (s, 2H), 6.12 (s, 1H), 5.27 (br s, 2H), 4.98 (br s, 1H), 4.11 (t, *J* = 4 Hz, 2H), 3.79 (m, 4H), 3.61 (m, 4H), 3.54 (br s, 1H), 3.43 (m, 3H), 1.65 (m, 6H), 1.38 (m, 4H), 1.13 (br s, 2H), 0.93 (t, *J* = 5 Hz, 6H). ^13^C-NMR (75.47 MHz, CDCl_3_) δ 174.67, 161.71, 161.37, 160.25, 158.00, 155.05, 120.35, 85.05, 64.55, 62.71, 50.83, 42.27, 39.24, 27.69, 26.51, 24.61, 23.66, 22.72, 20.88, 19.84.

### 3.2. Western Blot Analysis on Synthesized PI3K Inhibitors

#### General Methods

Analysis of PI3K inhibitors in C4-2 cells has been conducted essentially as previously described in Baiz et al. [33]. Briefly, cells were treated with compounds dissolved in DMSO or with solvent alone for 1 h. Then, cells were placed on ice and lysed in LSB buffer (20 mM HEPES, 150 mM NaCl, 1mM EDTA, 0.5% sodium deoxycholate, 1% Nonidet P-40, 1 mM DTT, pH 7.4) that contained protease inhibitors (10 μg/mL aprotinin, 10 μg/mL leupeptin, 10 μg/mL pepstatin, 1 mM benzamidine, 1 mM PMSF) and phosphatase inhibitors (1 mM NaVO_4_, 50 mM β-glycerophosphate, 40 mM *p*-nitrophenylphosphate, 40 mM NaF and 1 μg/mL microcystin). Cell lysates were centrifuged at 15,000 rpm for 15 min at 4 °C to remove nonsoluble material, and the supernatants equalized by protein content were separated on 12% SDS–PAGE gels and transferred onto a nitrocellulose or PVDF membrane (PerkinElmer, Waltham, MA, USA). Detection of pS473 was performed with rabbit polyclonal phospho-AKT (Ser473) (D9E) XP. Equal loading was controlled by staining membranes with Ponceau S and by probing the membranes with anti-AKT or anti-β-actin antibodies. AKT and pS473 antibodies were purchased from Cell Signaling Technology (Beverly, MA, USA), anti-β-actin antibodies and other reagents were purchased from Sigma-Aldrich (St. Louis, MO, USA). Secondary goat anti-mouse and goat anti-rabbit antibodies were from Thermo Fisher Scientific. Protein bands were quantified using ImageJ software (National Institutes of Health, Bethesda, MD, USA) [33].

## 4. Conclusions

In conclusion, we conducted the synthesis and pilot characterization of a new trisubstituted pyrimidine PI3K inhibitor (**14**) with increased potency compared to the well-characterized PI3K inhibitor ZSTK474. We also demonstrated that this new compound can be used to produce a latent prodrug selectively activated in prostate tumors that secretes prostate-specific antigen (PSA) protease. Thus, attachment of a leucine linker did not prevent inhibition of AKT phosphorylation and hence did not prevent the inhibition of PI3K by the modified inhibitor (**20**). The leucine linker remains on the prodrug after cleavage by PSA of an inhibitor peptide that prevents penetration of inactive prodrug into target cells [33]. Future experiments will examine the toxicity profile of the new PI3K inhibitor and will determine if prodrugs based on this compound would provide more potent inhibition of PI3K in prostate tumors that secrete PSA compared to currently used PI3K inhibitors.

## Supplementary Materials

Supplementary materials include spectral data for new compounds.
