# Supplementary material for: Synthesis and PI3 Kinase Inhibition Activity of Some Novel Trisubstituted Morpholinopyrimidines
Source: Molecules. 2018 Jul 10;23(7):1675. doi: 10.3390/molecules23071675 (PMC6100461; doi:10.3390/molecules23071675)
Supplement: Supplementary file 1 [file molecules-23-01675-s001.pdf]

Article

Synthesis and PI3 Kinase Inhibition Activity of Some Novel Trisubstituted MorpholinoPyrimidines

Emily W. Wright[2], Ronald A. Nelson, Jr.[2], Yue Huang[1], George Kulik[1,3*] and Mark E. Welker[2*]

[1]Department of Cancer Biology and Comprehensive Cancer Center, Wake Forest School of Medicine, Medical Center Blvd., Winston-Salem, NC 27157, USA
[2]Department of Chemistry, Wake Forest University, Winston-Salem, NC 27109, USA
[3] Life Sciences program, College of Science, Alfaisal University, Riyadh 11533, Saudi Arabia.
*To whom correspondence should be addressed.
George Kulik, DVM, PhD. Department of Cancer Biology and Comprehensive Cancer Center Wake Forest University School of Medicine, Medical Center Blvd., Winston-Salem, NC 27127-1083, USA. Tel.: 336-713-7650. E-mail: gkulik@wakehealth.edu

Mark E Welker, PhD. Department of Chemistry, Wake Forest University, P.O. Box 7486, Winston-Salem, NC 27109, USA. Tel.: 336-758-5758. E-mail: welker@wfu.edu

Supplementary Material: Spectral data for new compounds reported.

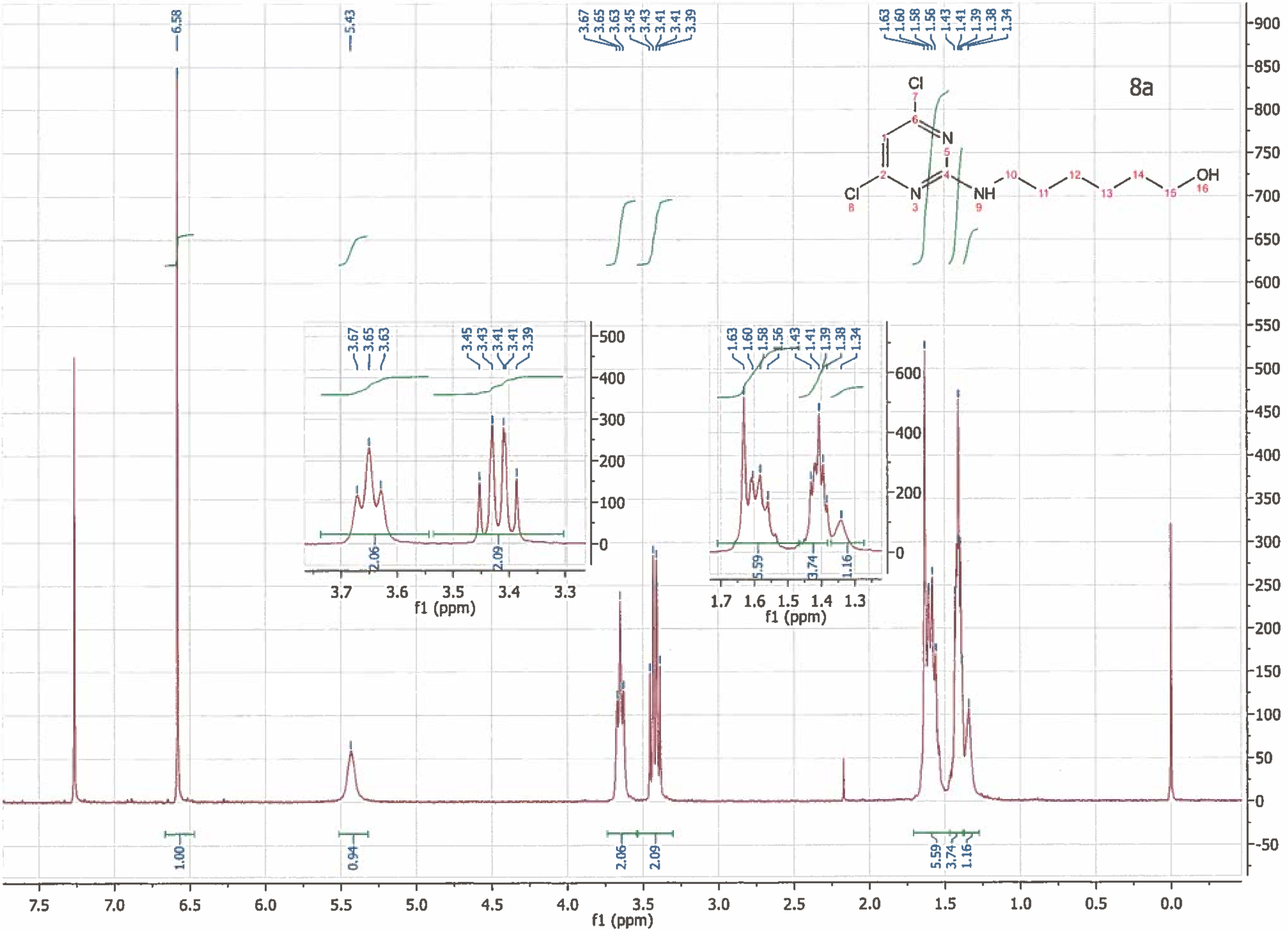

8a
6.58
5.43
3.67
3.65
3.63
3.45
3.43
3.41
3.41
3.39
1.63
1.60
1.58
1.56
1.43
1.41
1.39
1.38
1.34
1.00
0.94
2.06
2.09
5.59
3.74
1.16
f1 (ppm)

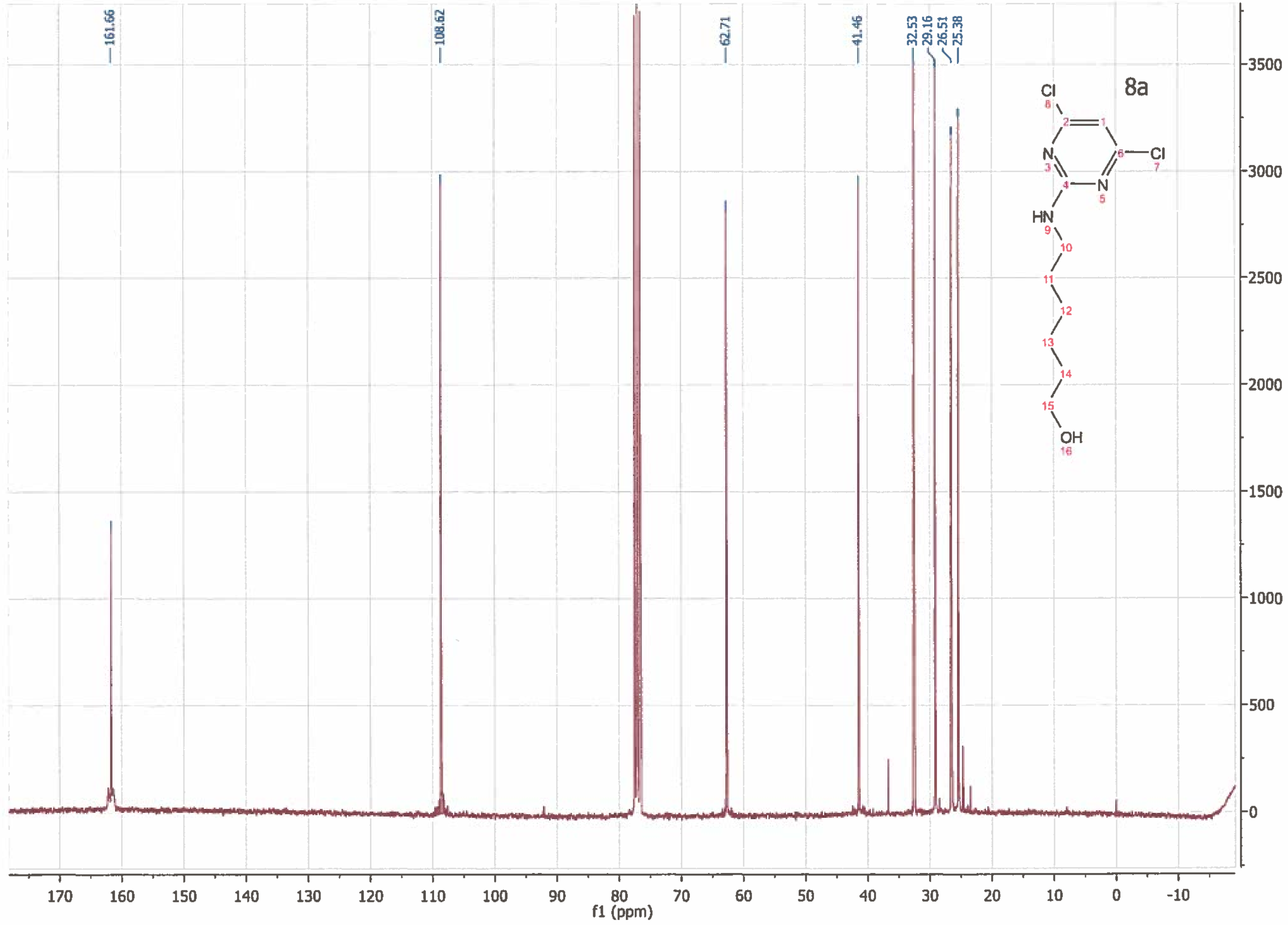
161.66
108.62
62.71
41.46
32.53
29.16
26.51
25.38
8a
Cl
N
HN
OH
f1 (ppm)

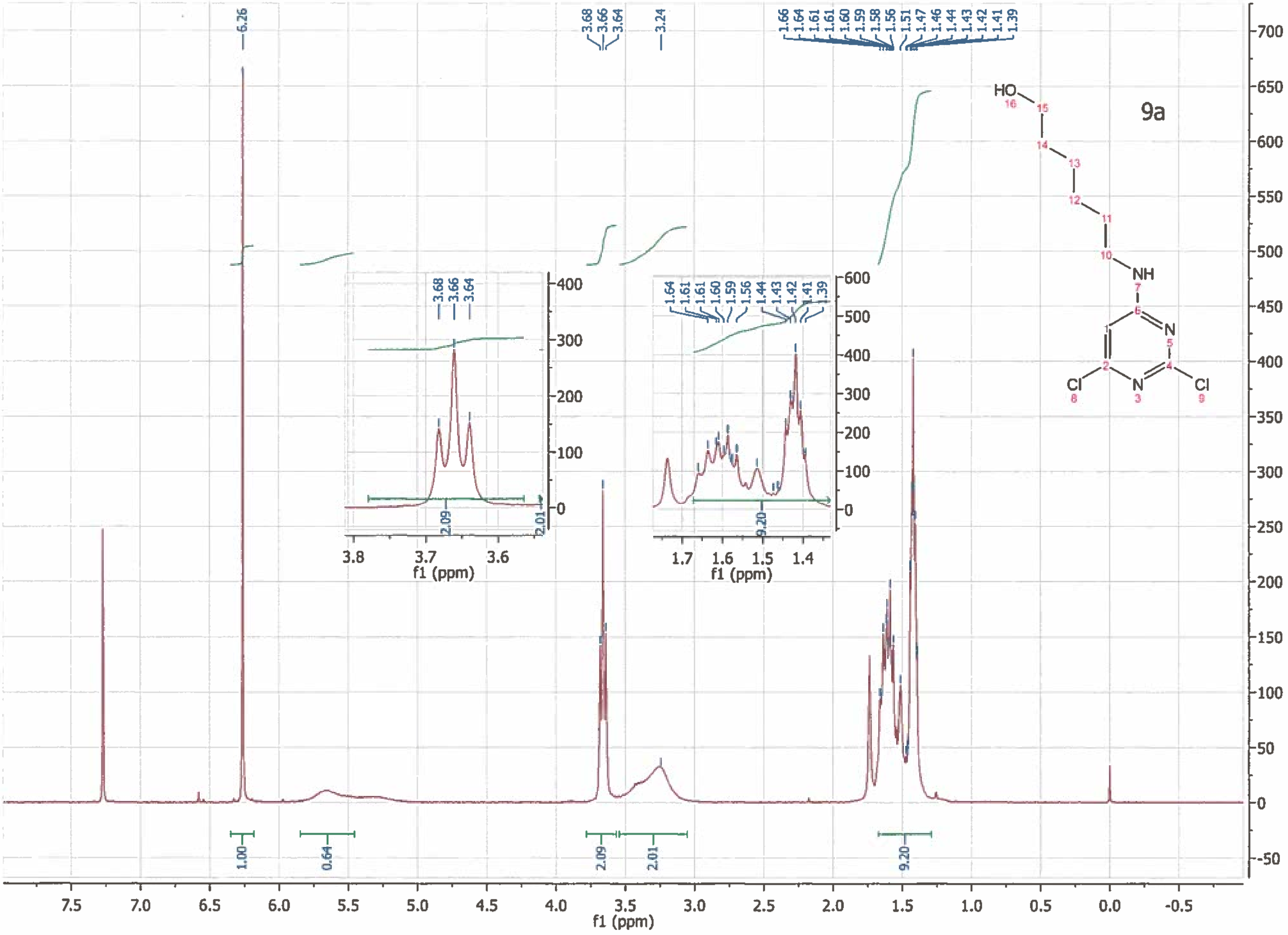

9a
HO
NH
N
N
Cl
Cl
6.26
3.68
3.66
3.64
3.24
1.66
1.64
1.61
1.61
1.60
1.59
1.58
1.56
1.51
1.47
1.46
1.44
1.43
1.42
1.41
1.39
1.00
0.64
2.09
2.01
9.20
f1 (ppm)

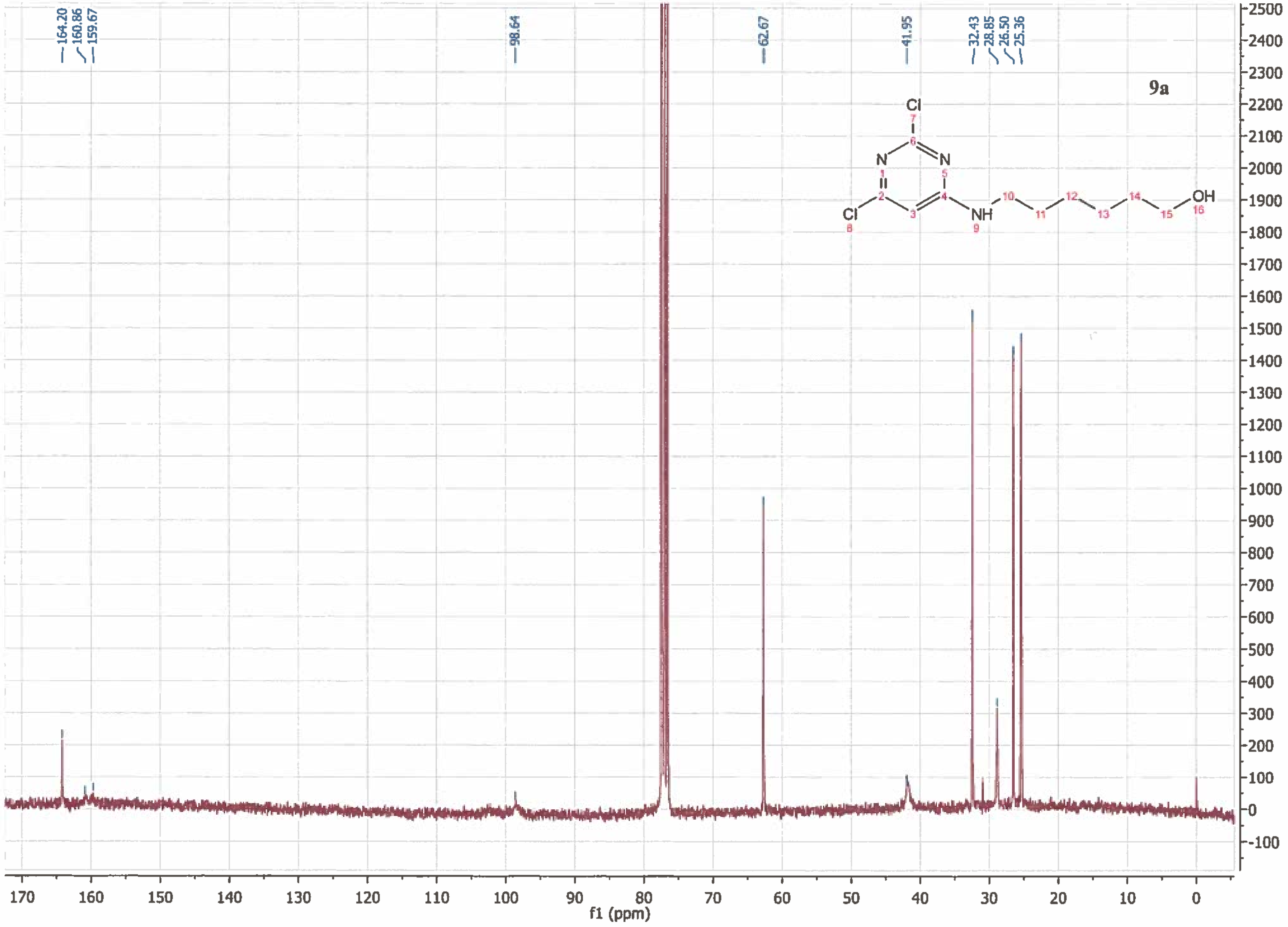

164.20
160.86
159.67
98.64
62.67
41.95
32.43
28.85
26.50
25.36
9a
Cl
N
N
Cl
NH
OH
f1 (ppm)

(4-(((4,6-dichloropyrimidin-2-yl) amino) methyl) phenyl) methanol, 8b.

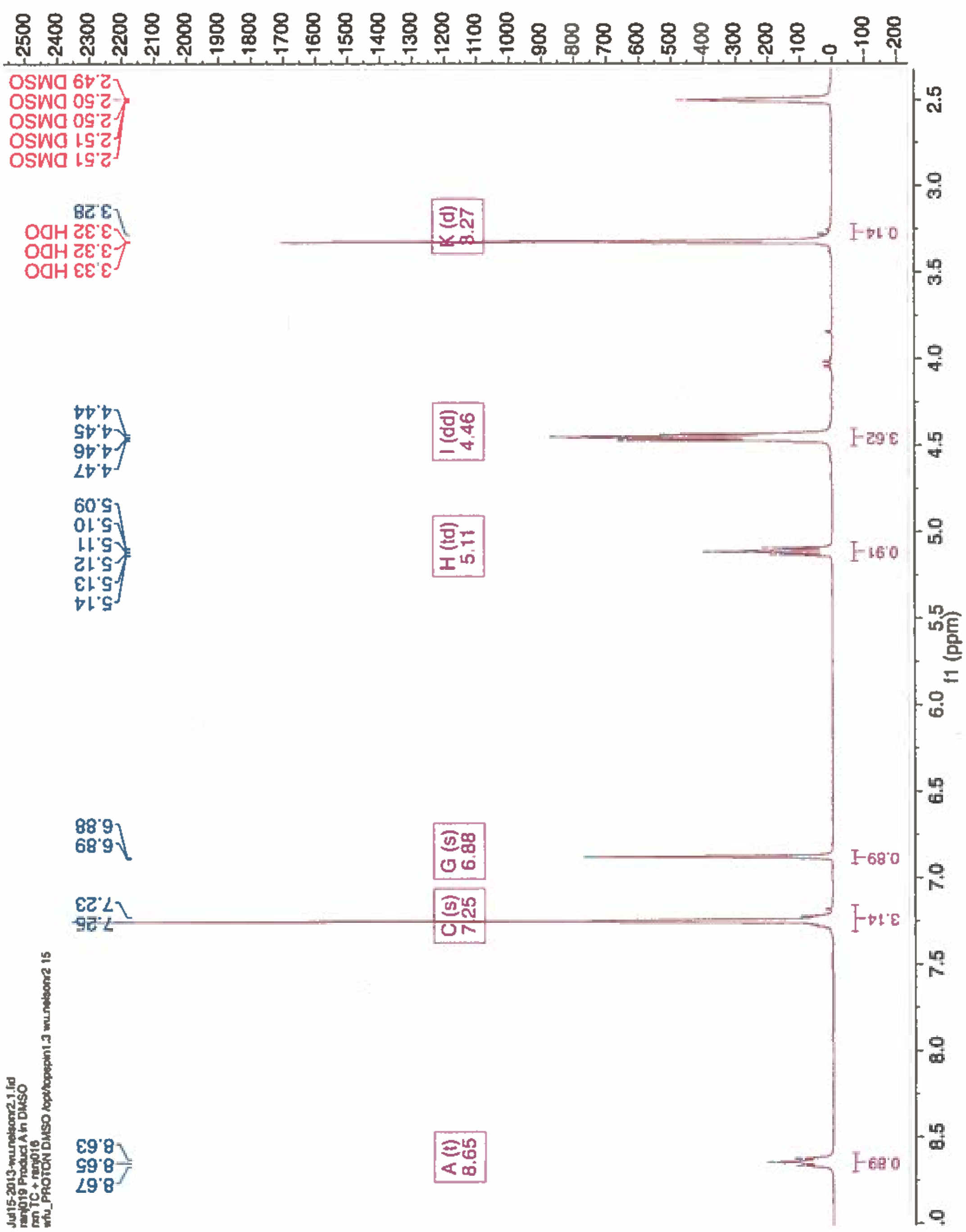

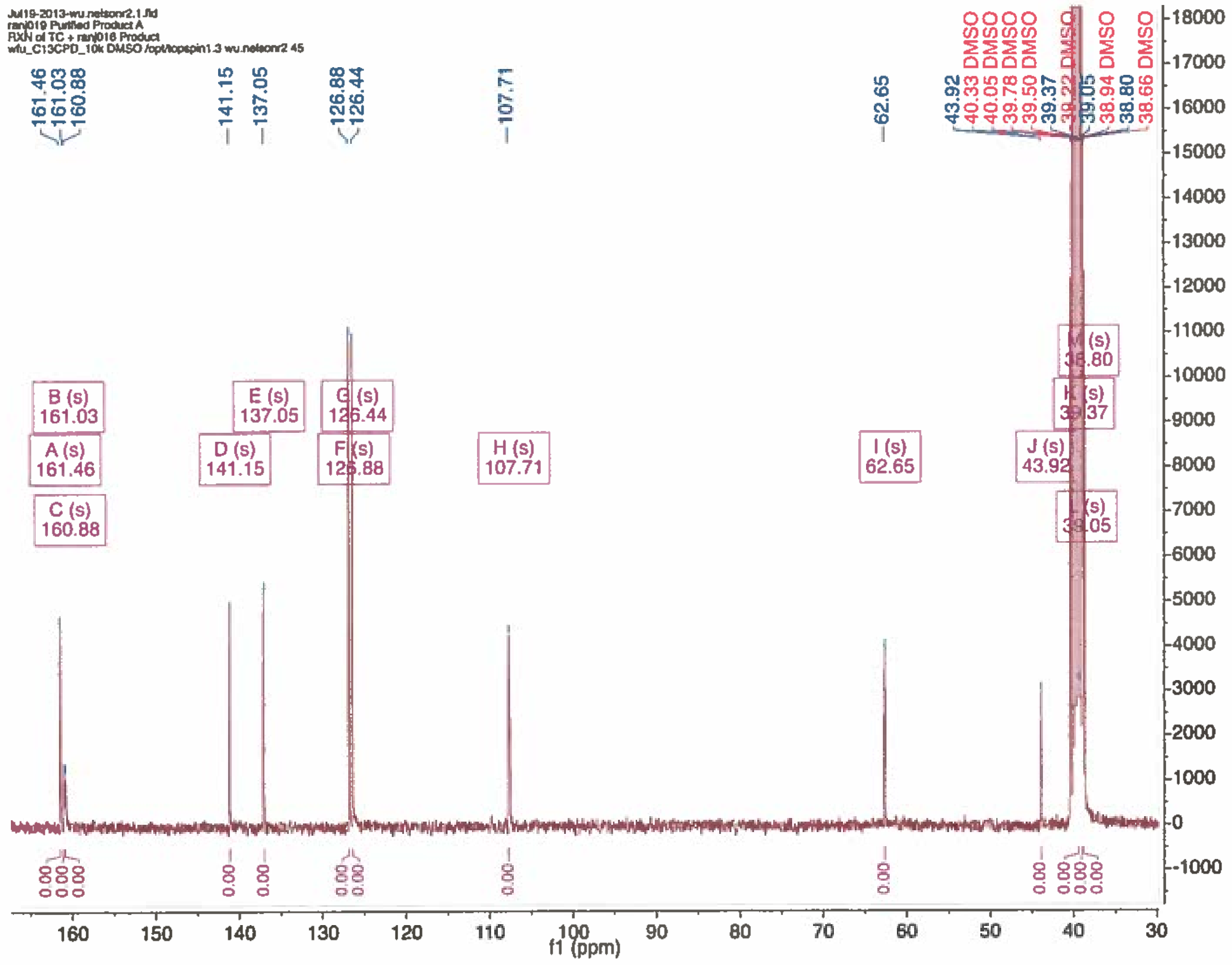
Jul19-2013-wu.nelsonr2.1.fid
ranj019 Purified Product A
RXN of TC + ranj016 Product
wfu_C13CPD_10k DMSO /opt/topspin1.3 wu.nelsonr2 45
161.46
161.03
160.88
141.15
137.05
126.88
126.44
107.71
62.65
43.92
40.33 DMSO
40.05 DMSO
39.78 DMSO
39.50 DMSO
39.37
39.22 DMSO
39.05
38.94 DMSO
38.80
38.66 DMSO
B (s) 161.03
A (s) 161.46
C (s) 160.88
D (s) 141.15
E (s) 137.05
G (s) 126.44
F (s) 126.88
H (s) 107.71
I (s) 62.65
J (s) 43.92
f1 (ppm)

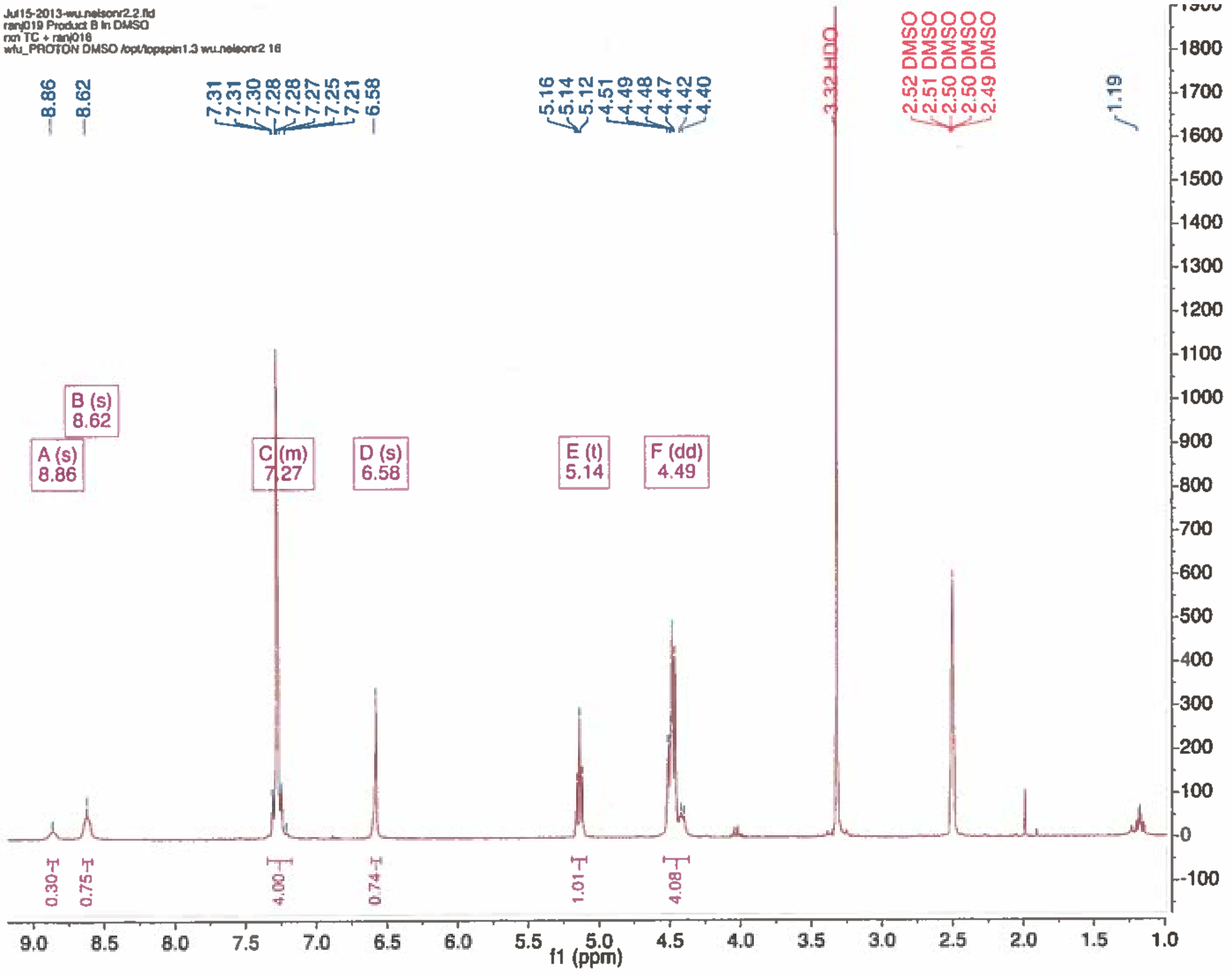

(4-(((2,6-dichloropyrimidin-4-yl)amino)methyl)phenyl)methanol, 9b.

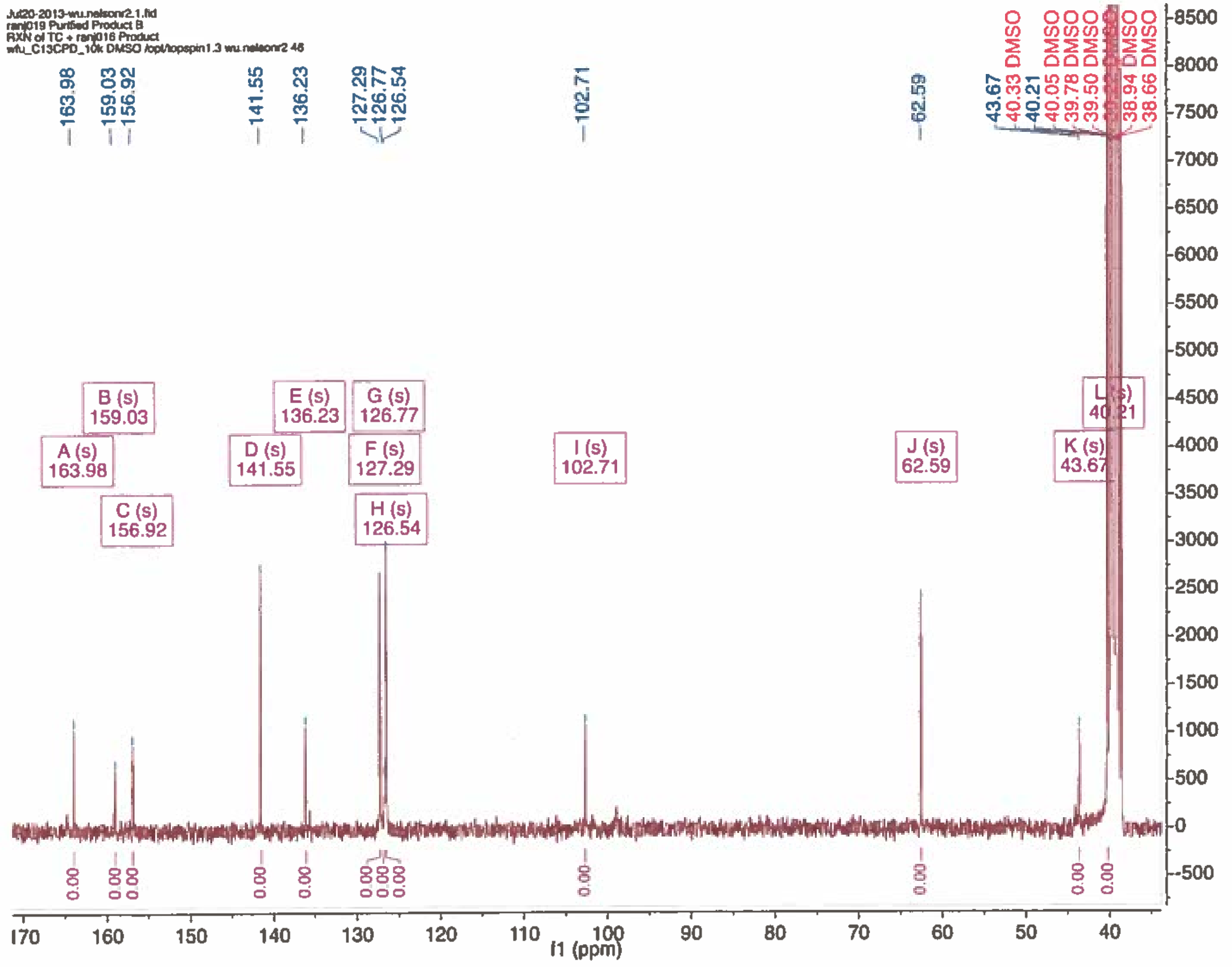
Jul20-2013-wu.nelsonr2.1.fid
ranj019 Purified Product B
RXN of TC + ranj016 Product
wfu_C13CPD_10k DMSO /opt/topspin1.3 wu.nelsonr2 46
163.98
159.03
156.92
141.55
136.23
127.29
126.77
126.54
102.71
62.59
43.67
40.33 DMSO
40.21
40.05 DMSO
39.78 DMSO
39.50 DMSO
38.94 DMSO
38.66 DMSO
A (s) 163.98
B (s) 159.03
C (s) 156.92
D (s) 141.55
E (s) 136.23
F (s) 127.29
G (s) 126.77
H (s) 126.54
I (s) 102.71
J (s) 62.59
f1 (ppm)

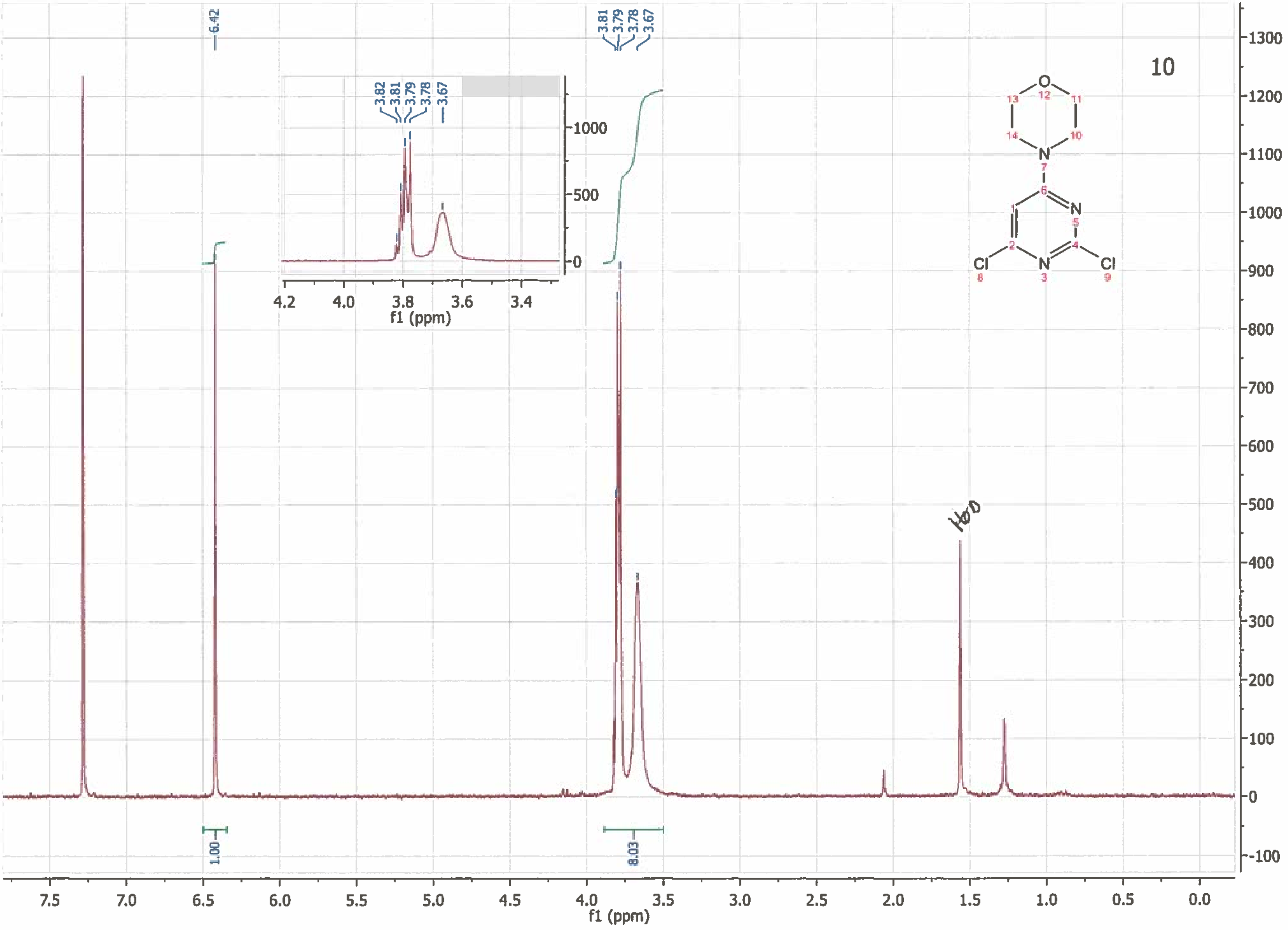

10
6.42
3.81
3.79
3.78
3.67
3.82
3.81
3.79
3.78
3.67
1.00
8.03
f1 (ppm)
f1 (ppm)
Cl
N
Cl
N
N
N
O
H₂O

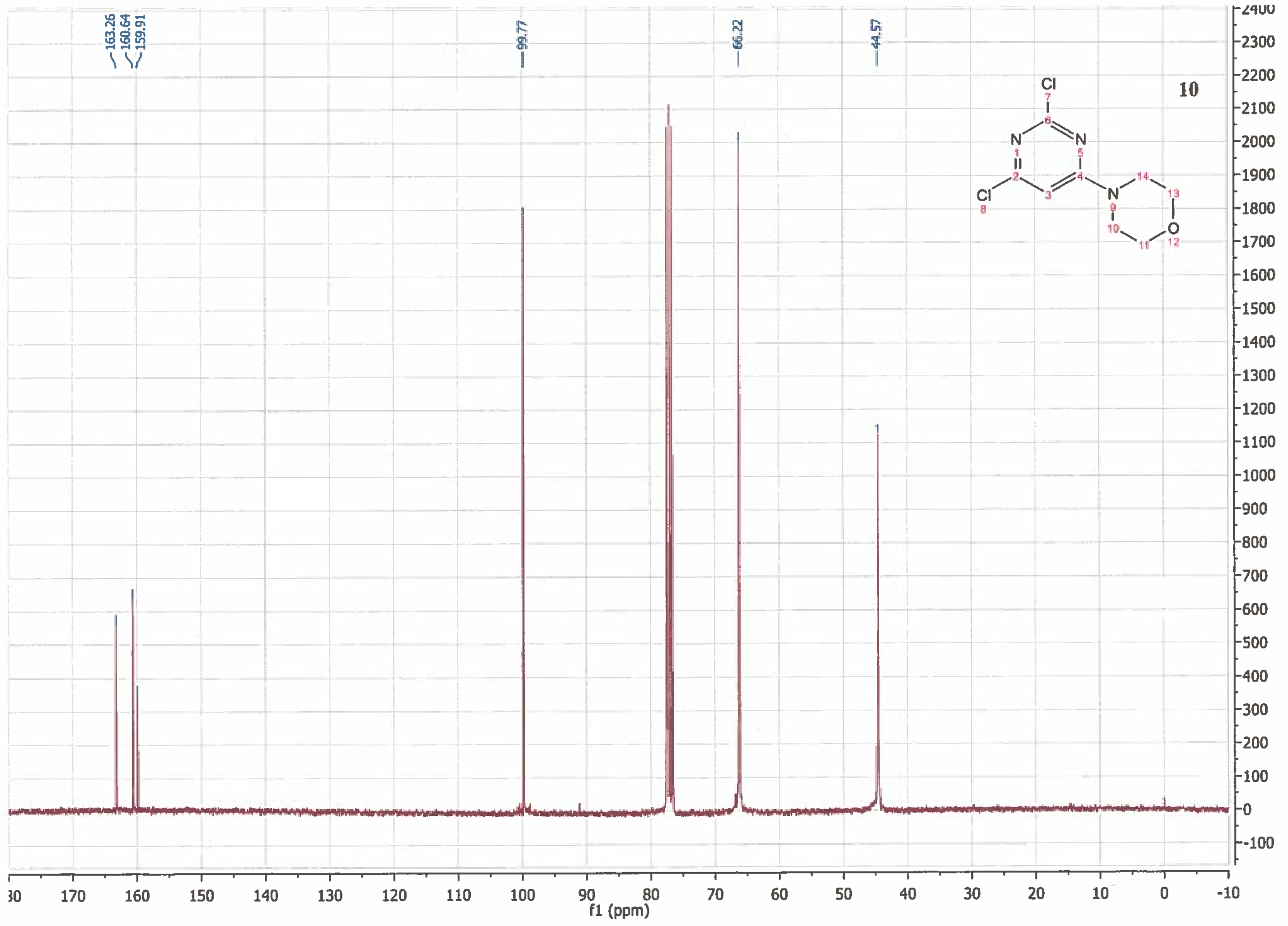

163.26
160.64
159.91
99.77
66.22
44.57
Cl
N
N
Cl
N
O
10
f1 (ppm)

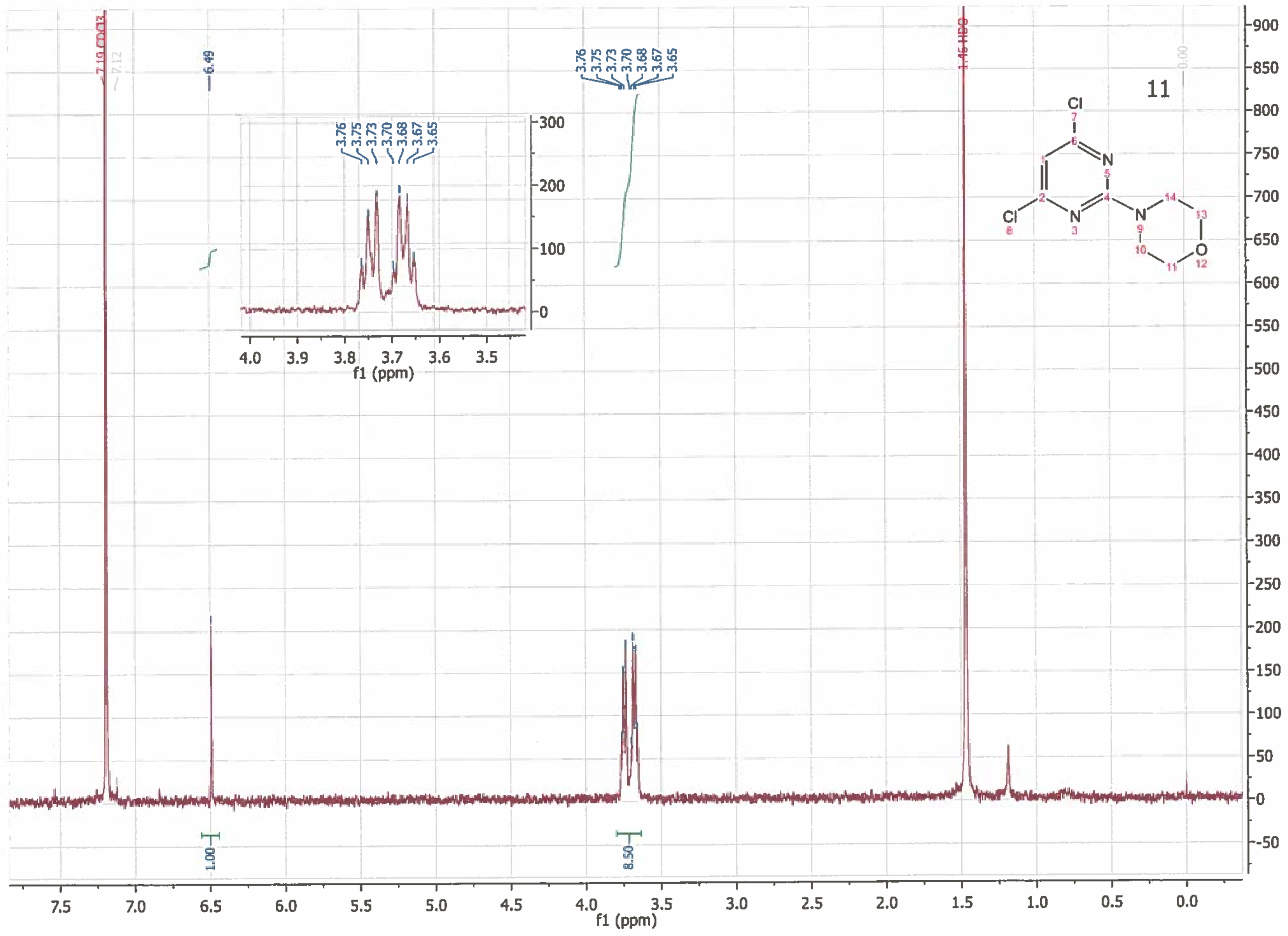
7.19 CDCl3
7.12
6.49
3.76
3.75
3.73
3.70
3.68
3.67
3.65
1.45 HDO
0.00
11
1.00
8.50
f1 (ppm)

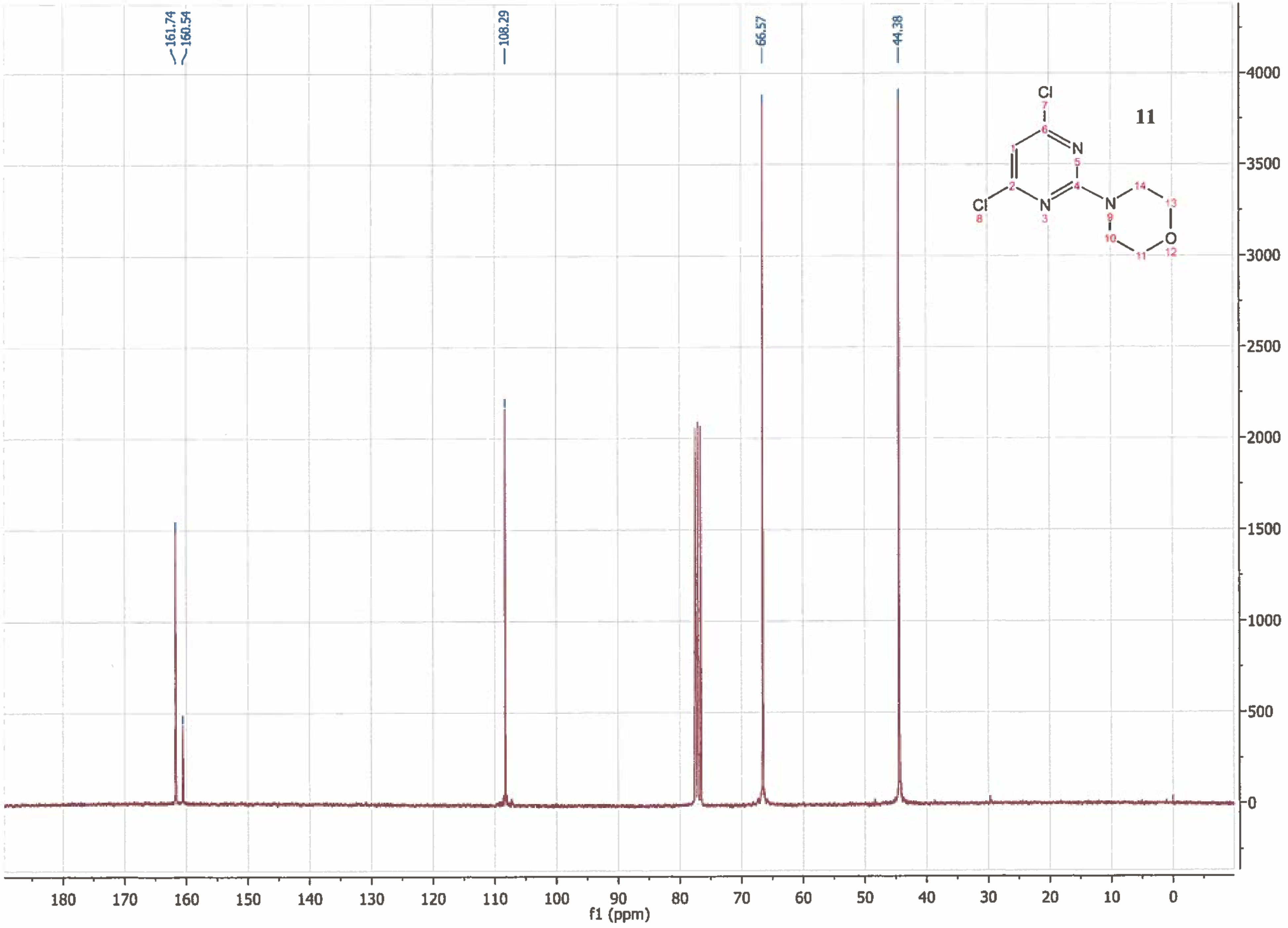

161.74
160.54
108.29
66.57
44.38
Cl
N
N
Cl
N
O
11
4000
3500
3000
2500
2000
1500
1000
500
0
180
170
160
150
140
130
120
110
100
90
80
70
60
50
40
30
20
10
0
f1 (ppm)

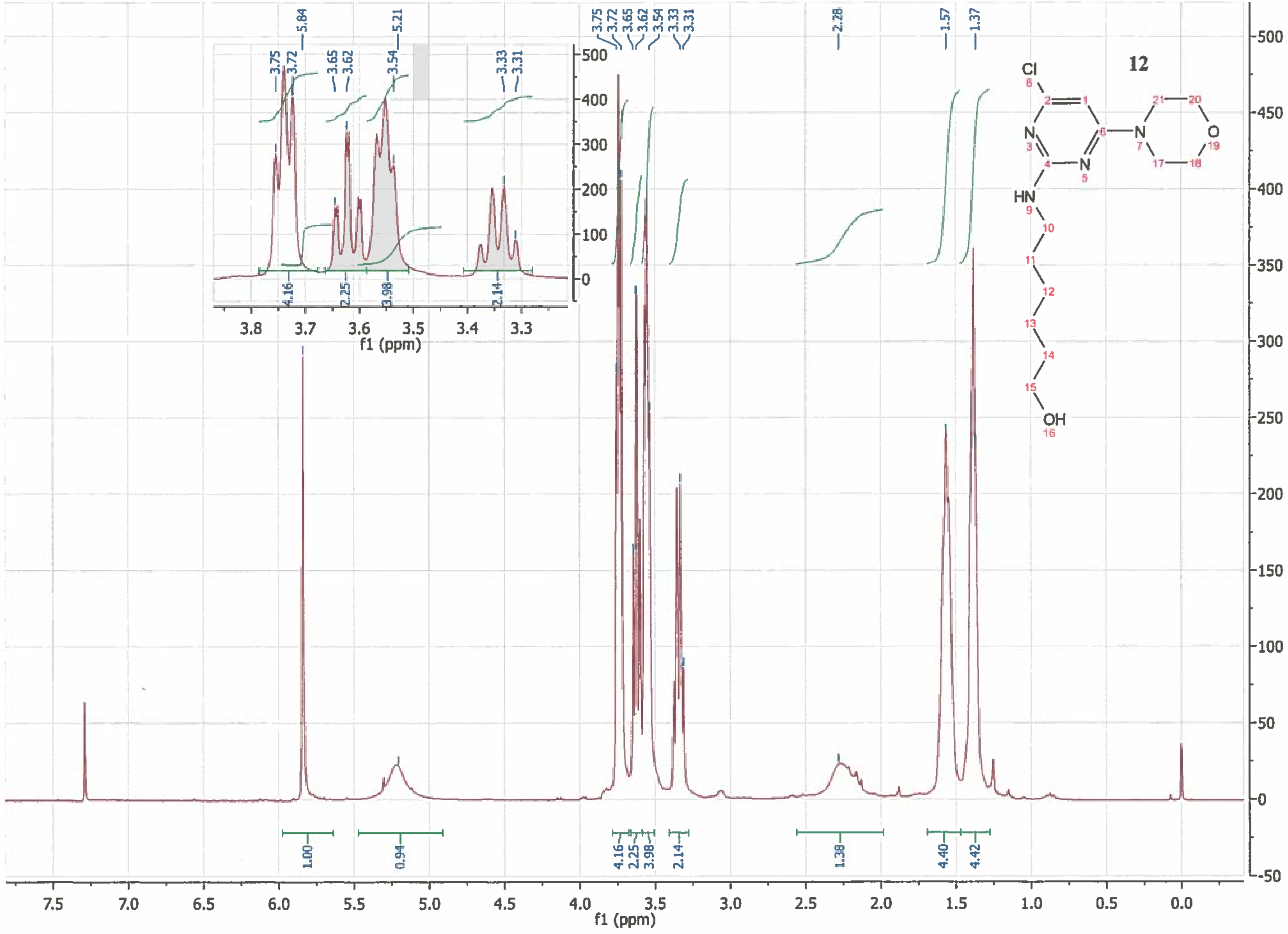

12
Cl
HN
OH
N
N
N
O
f1 (ppm)
3.75
3.72
3.65
3.62
3.54
3.33
3.31
2.28
1.57
1.37
5.84
5.21
1.00
0.94
4.16
2.25
3.98
2.14
1.38
4.40
4.42

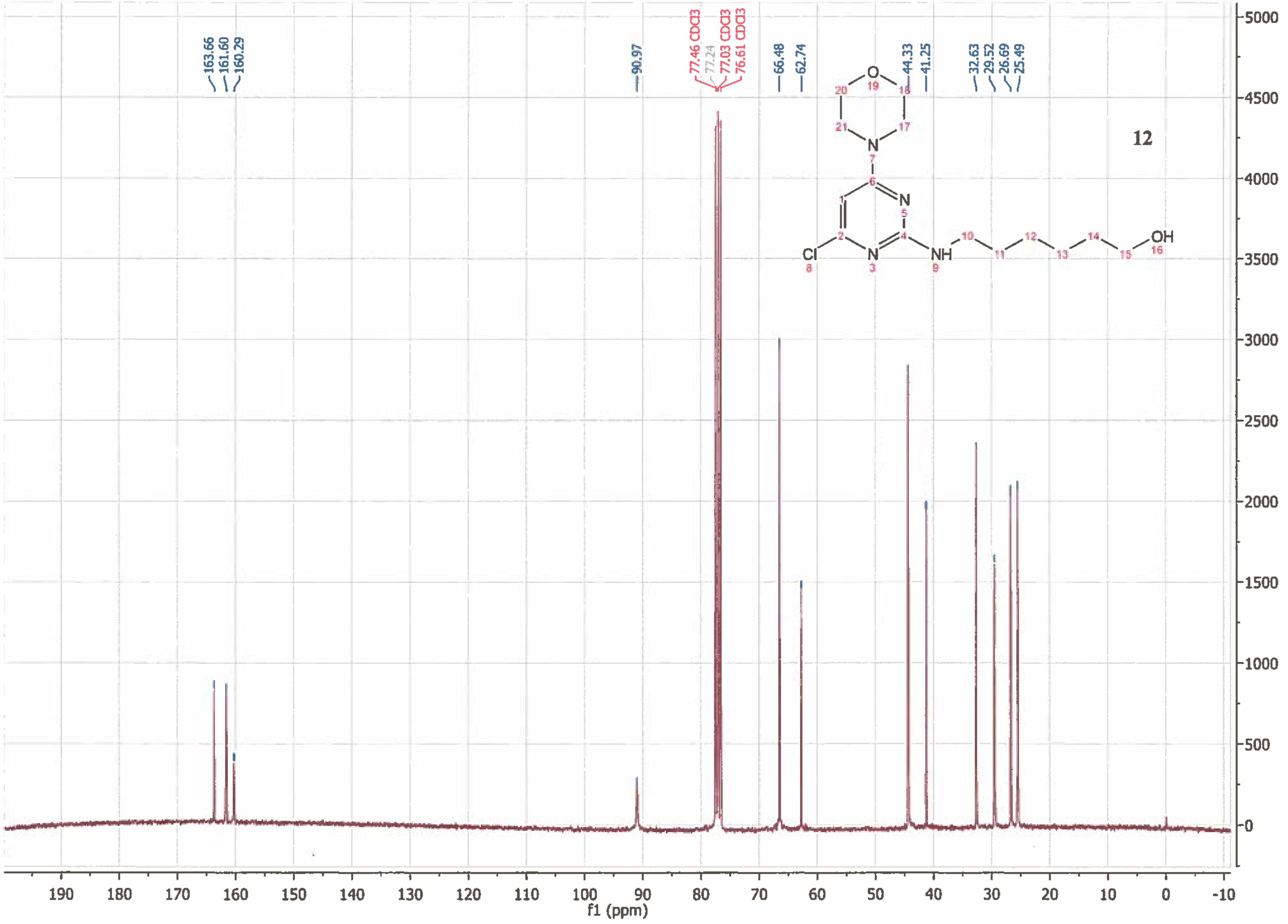
163.66
161.60
160.29
90.97
77.46 CDCl3
77.24
77.03 CDCl3
76.61 CDCl3
66.48
62.74
44.33
41.25
32.63
29.52
26.69
25.49
12
f1 (ppm)

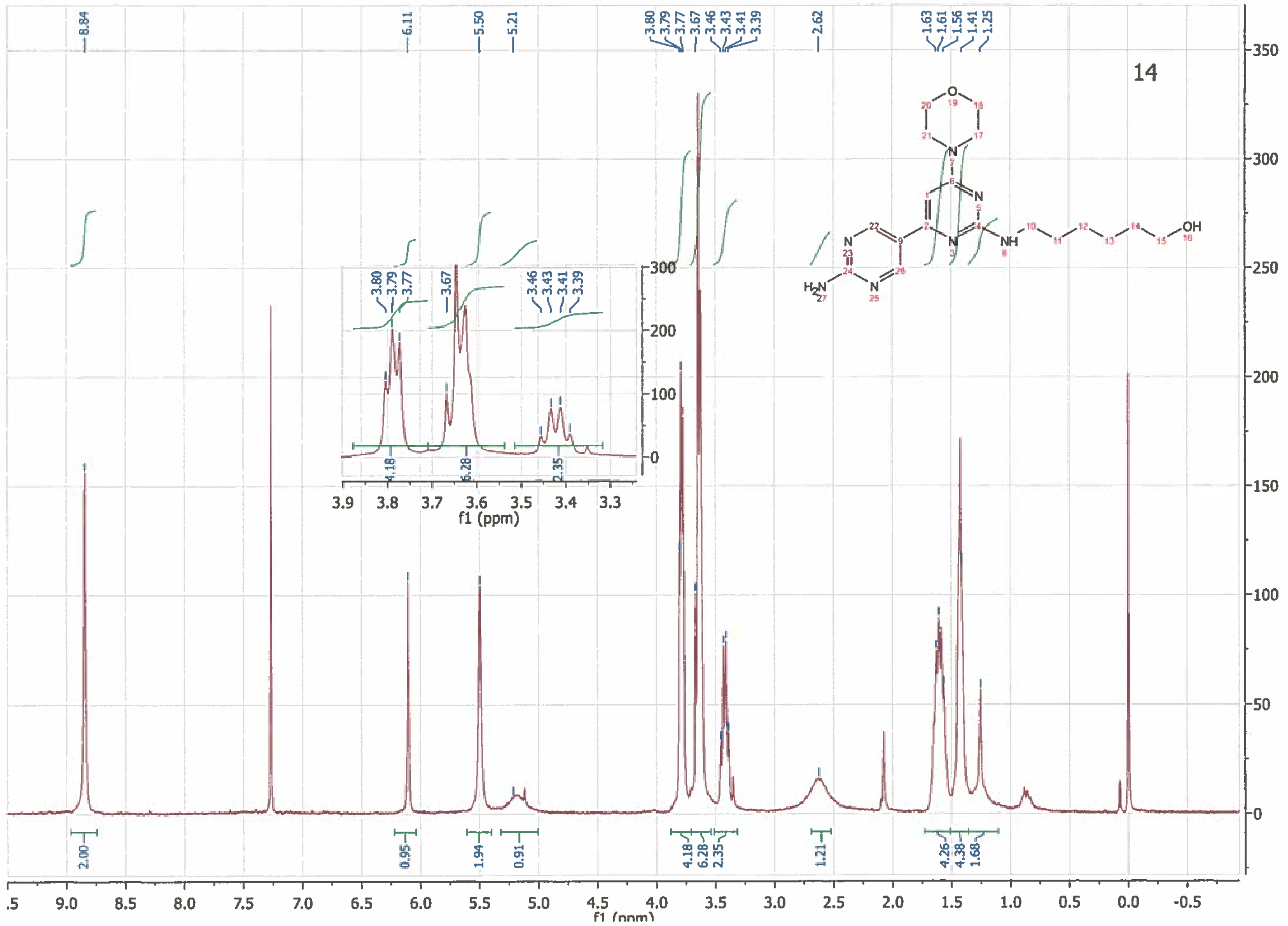
14
8.84
6.11
5.50
5.21
3.80
3.79
3.77
3.67
3.46
3.43
3.41
3.39
2.62
1.63
1.61
1.56
1.41
1.25
2.00
0.95
1.94
0.91
4.18
6.28
2.35
1.21
4.26
4.38
1.68
f1 (ppm)
NH
OH
H_2N

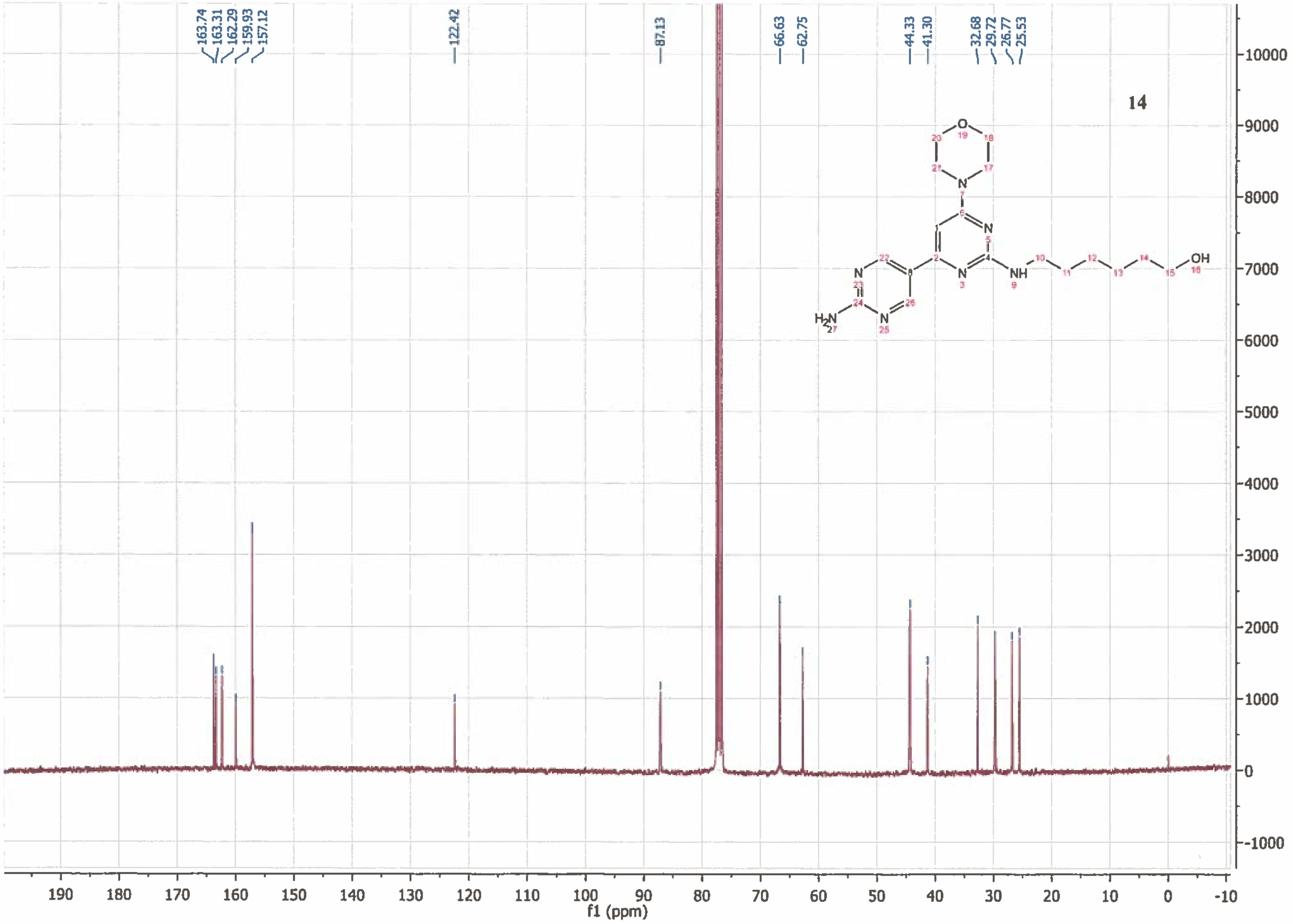

163.74
163.31
162.29
159.93
157.12
122.42
87.13
66.63
62.75
44.33
41.30
32.68
29.72
26.77
25.53
14
O
N
NH
OH
H2N
f1 (ppm)

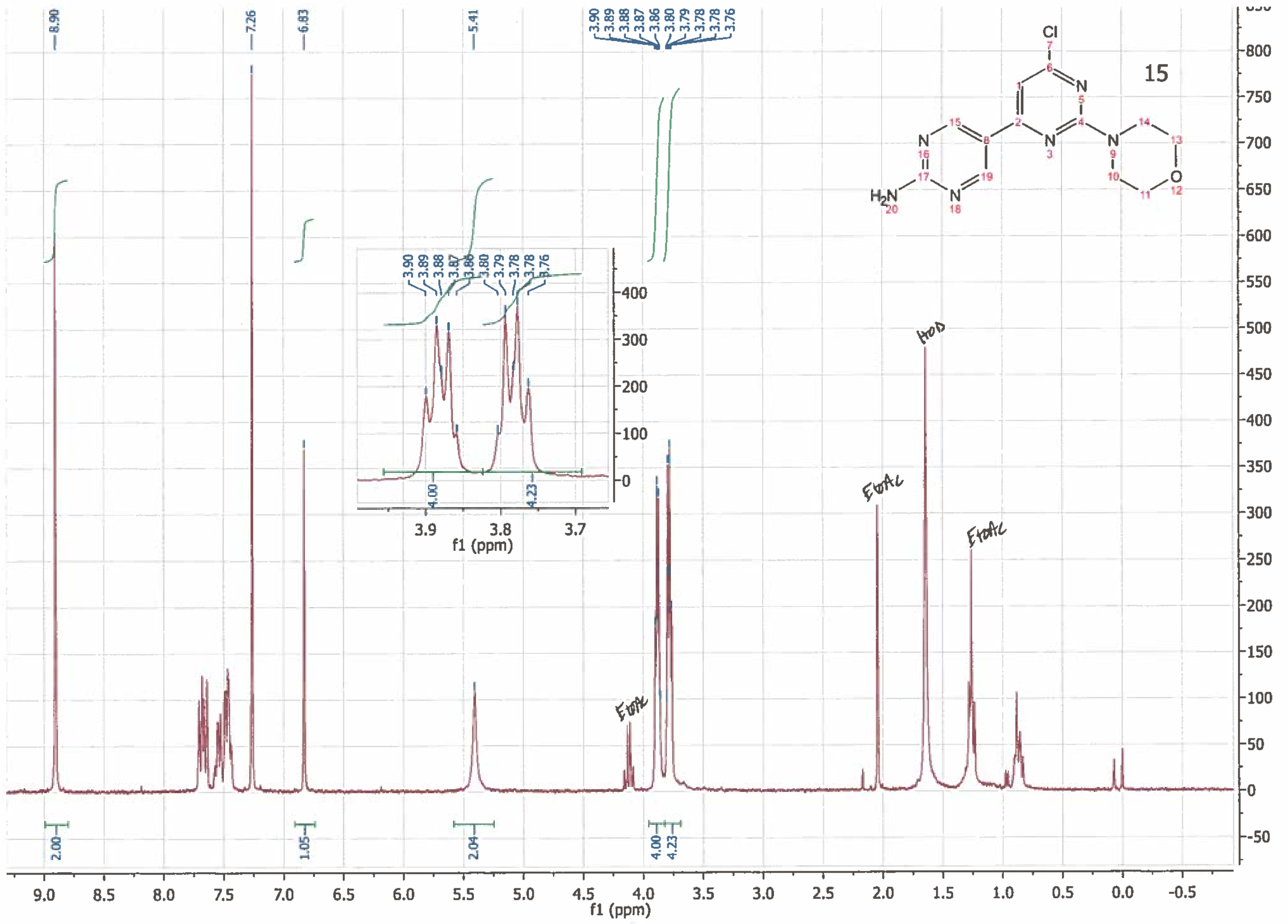
8.90
7.26
6.83
5.41
3.90
3.89
3.88
3.87
3.86
3.80
3.79
3.78
3.78
3.76
2.00
1.05
2.04
4.00
4.23
f1 (ppm)
EtOAc
HOD
15
Cl
H2N

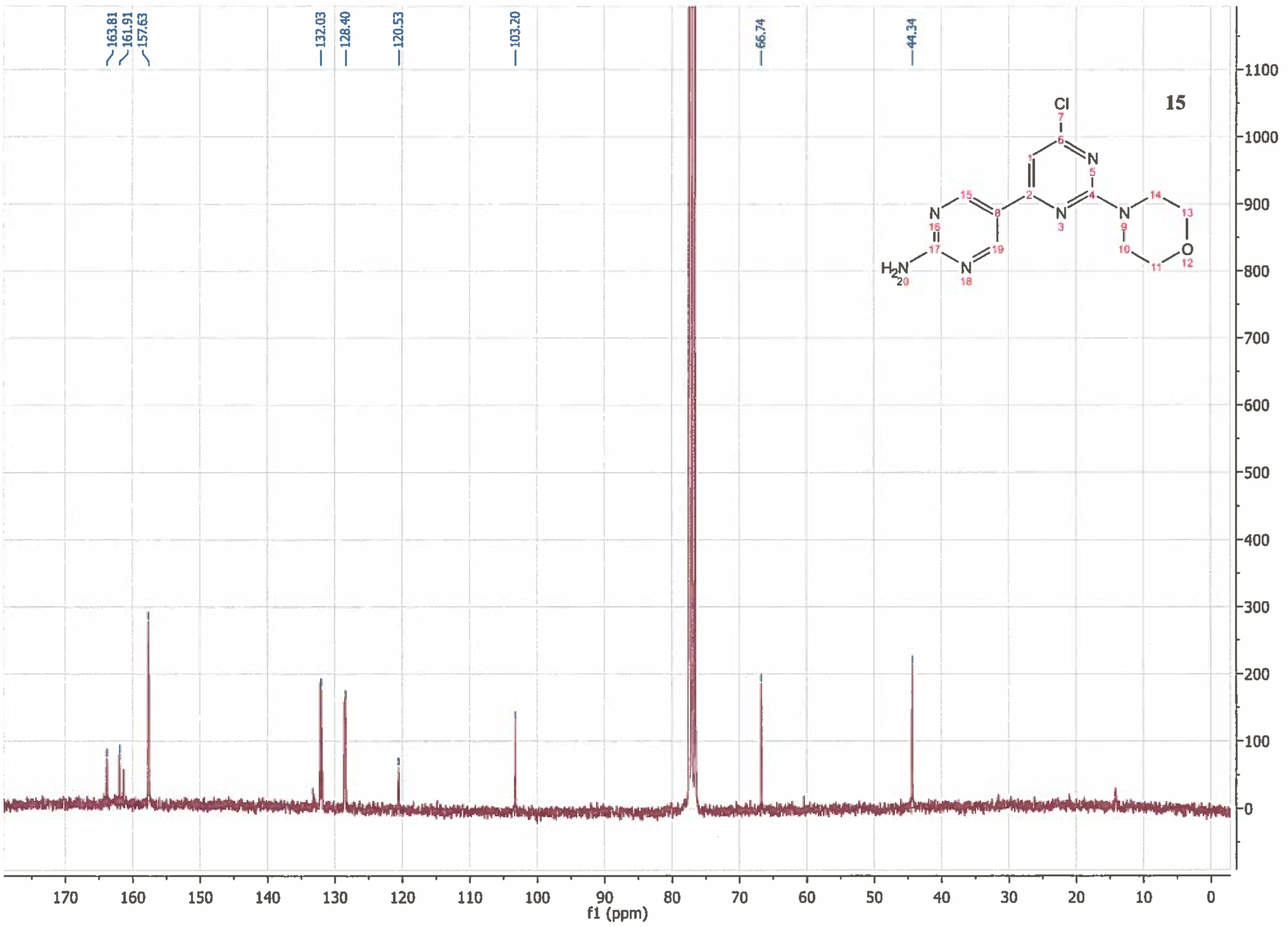
163.81
161.91
157.63
132.03
128.40
120.53
103.20
66.74
44.34
15
Cl
N
N
N
N
N
H_2N
O
170
160
150
140
130
120
110
100
90
80
70
60
50
40
30
20
10
0
f1 (ppm)
1100
1000
900
800
700
600
500
400
300
200
100
0

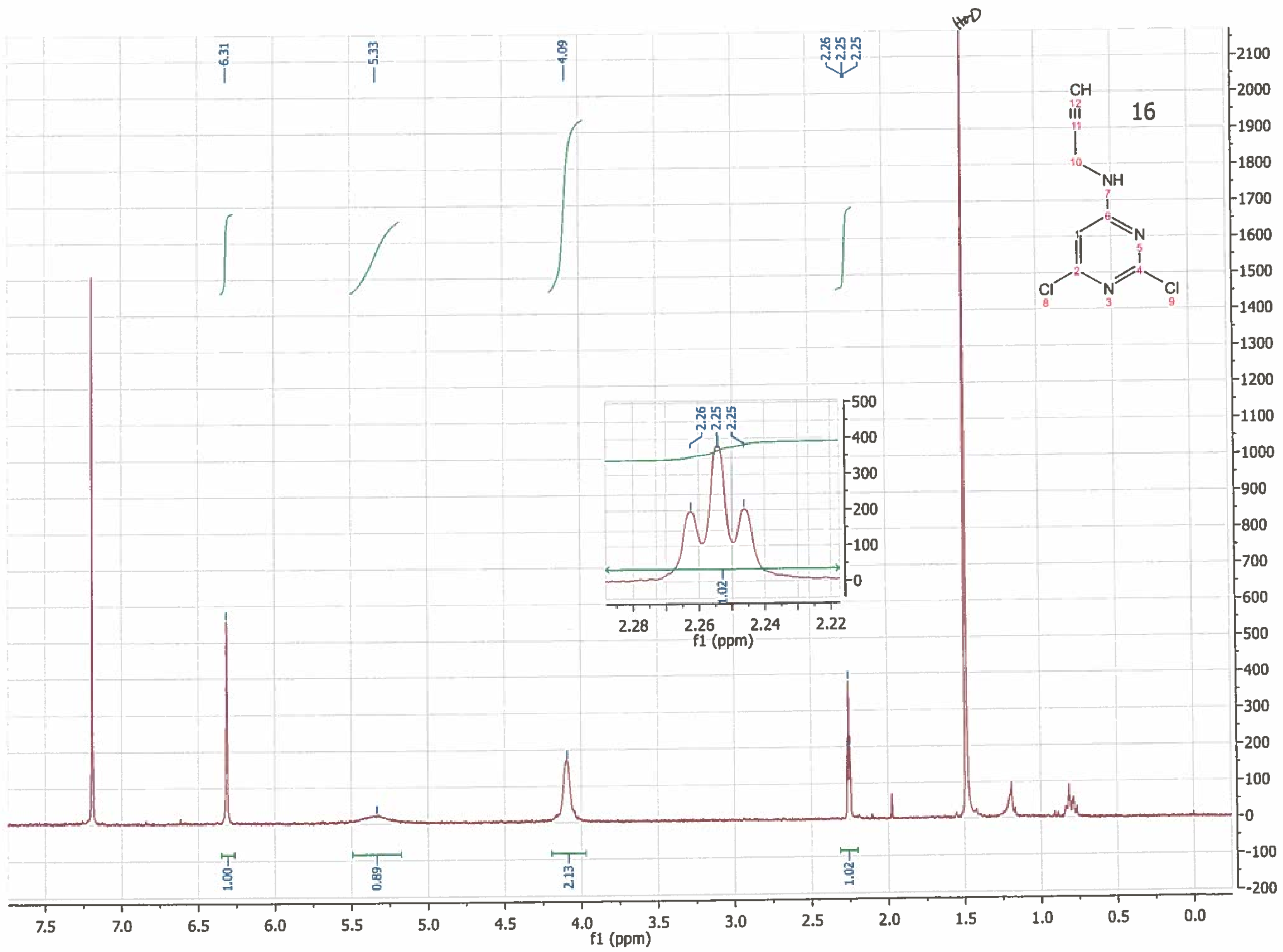

6.31
5.33
4.09
2.26
2.25
2.25
H2O
16
CH
NH
N
Cl
1.00
0.89
2.13
1.02
f1 (ppm)

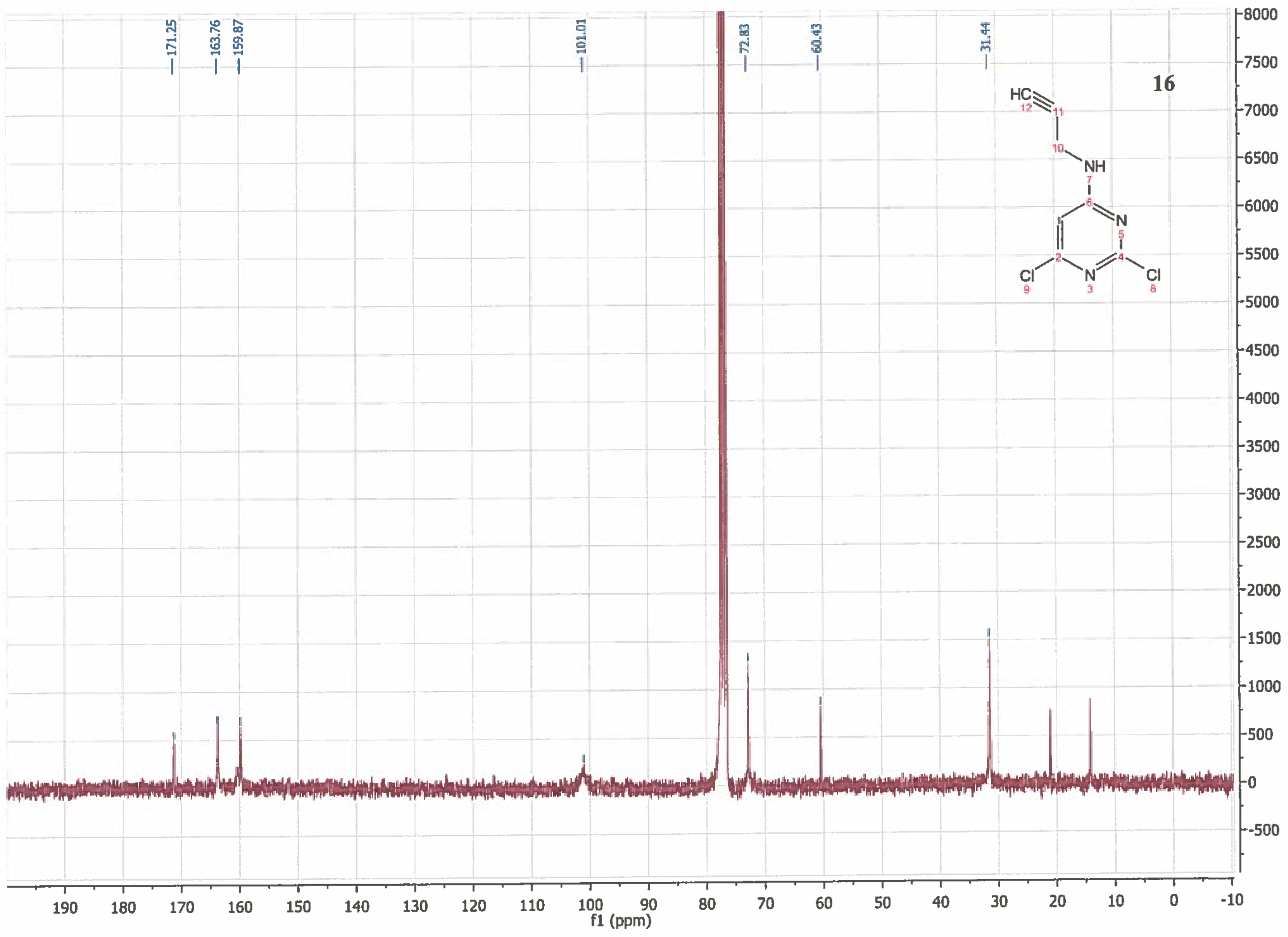
171.25
163.76
159.87
101.01
72.83
60.43
31.44
HC
NH
N
Cl
Cl
N
16
f1 (ppm)

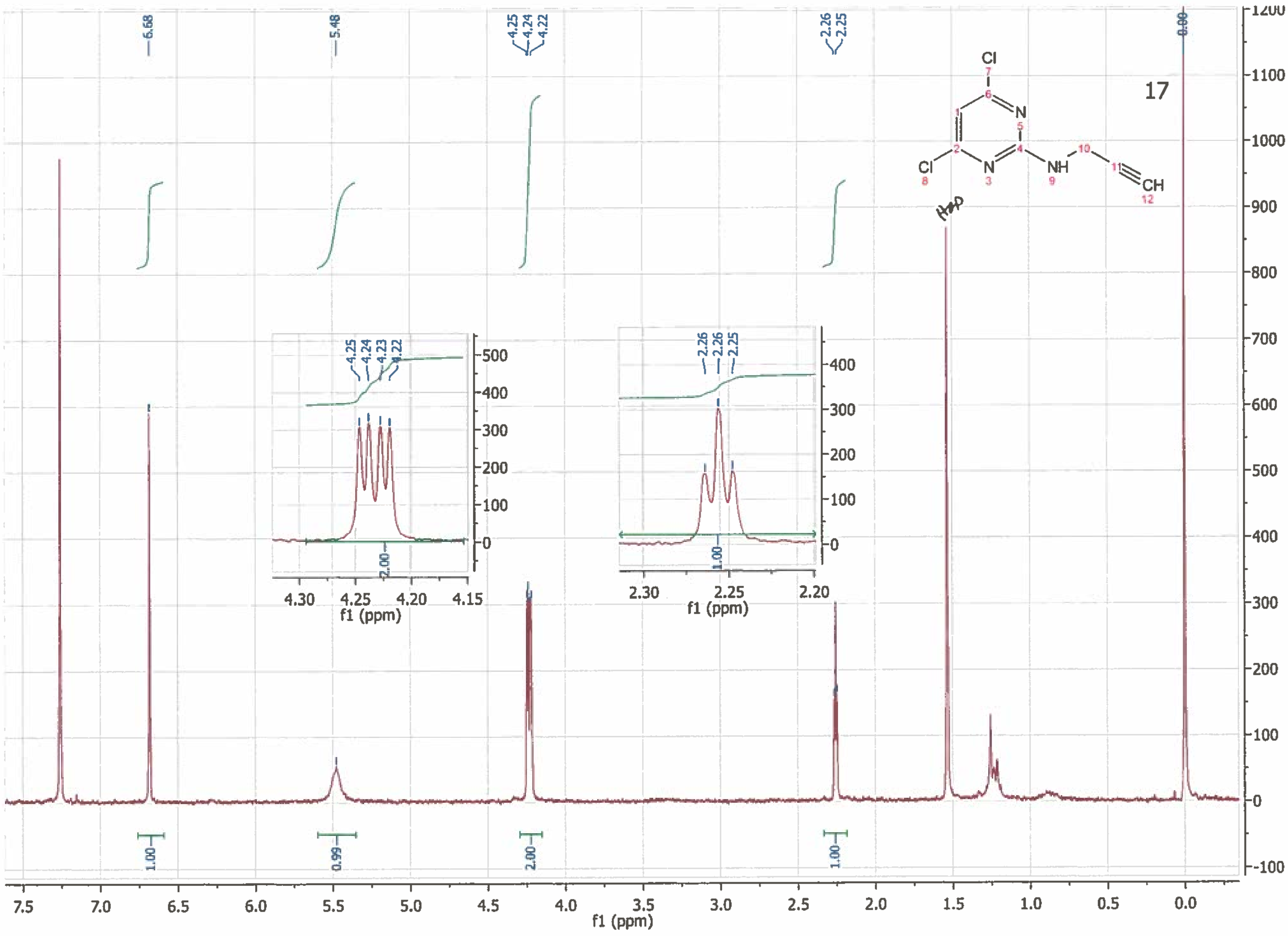

6.68
5.48
4.25
4.24
4.22
2.26
2.25
0.00
17
Cl
N
Cl
N
NH
CH
H2O
4.25
4.24
4.23
4.22
2.00
4.30
4.25
4.20
4.15
f1 (ppm)
2.26
2.26
2.25
1.00
2.30
2.25
2.20
f1 (ppm)
1.00
0.99
2.00
1.00
f1 (ppm)

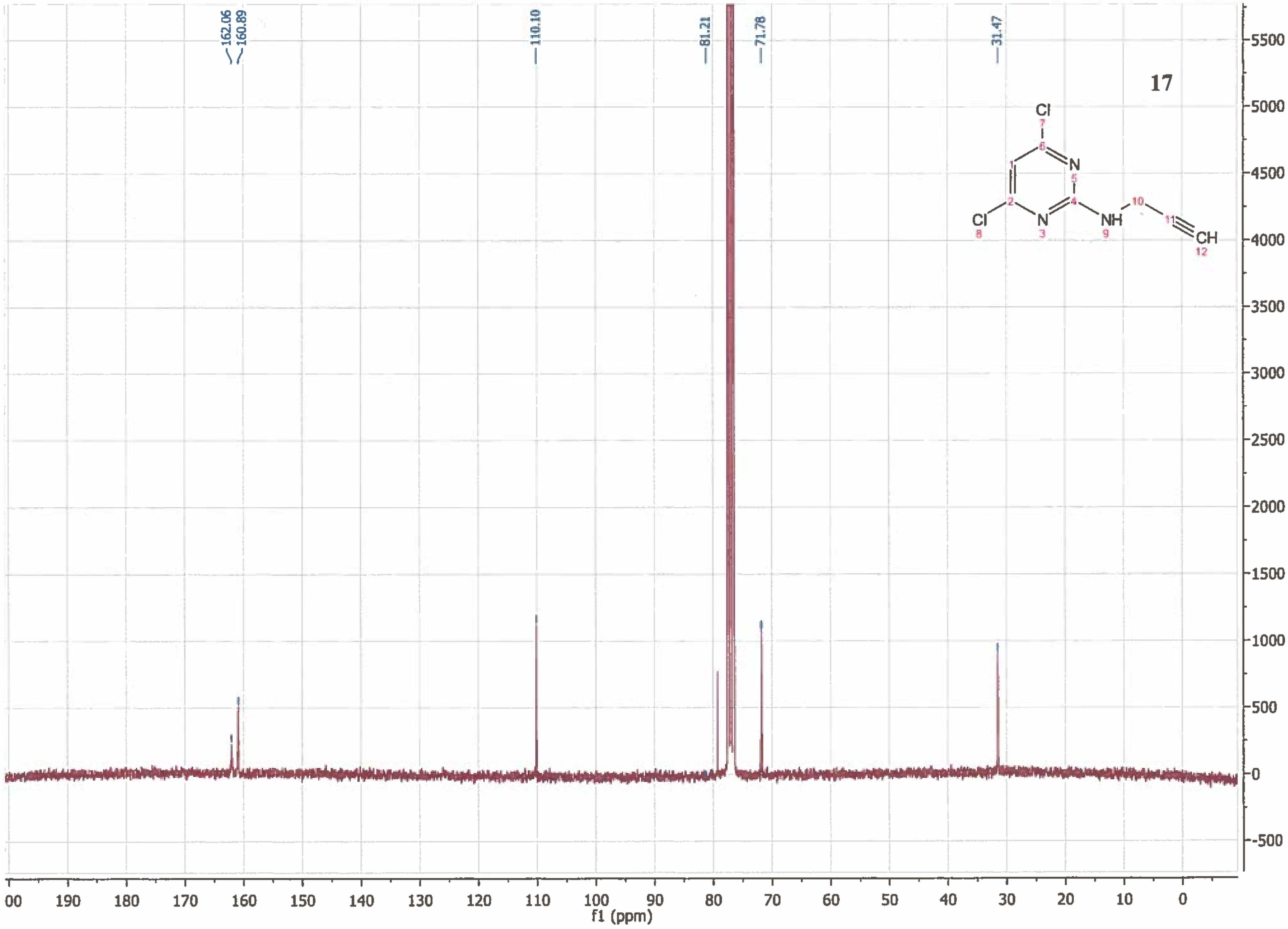
162.06
160.89
110.10
81.21
71.78
31.47
17
Cl
N
NH
CH
Cl
N
f1 (ppm)

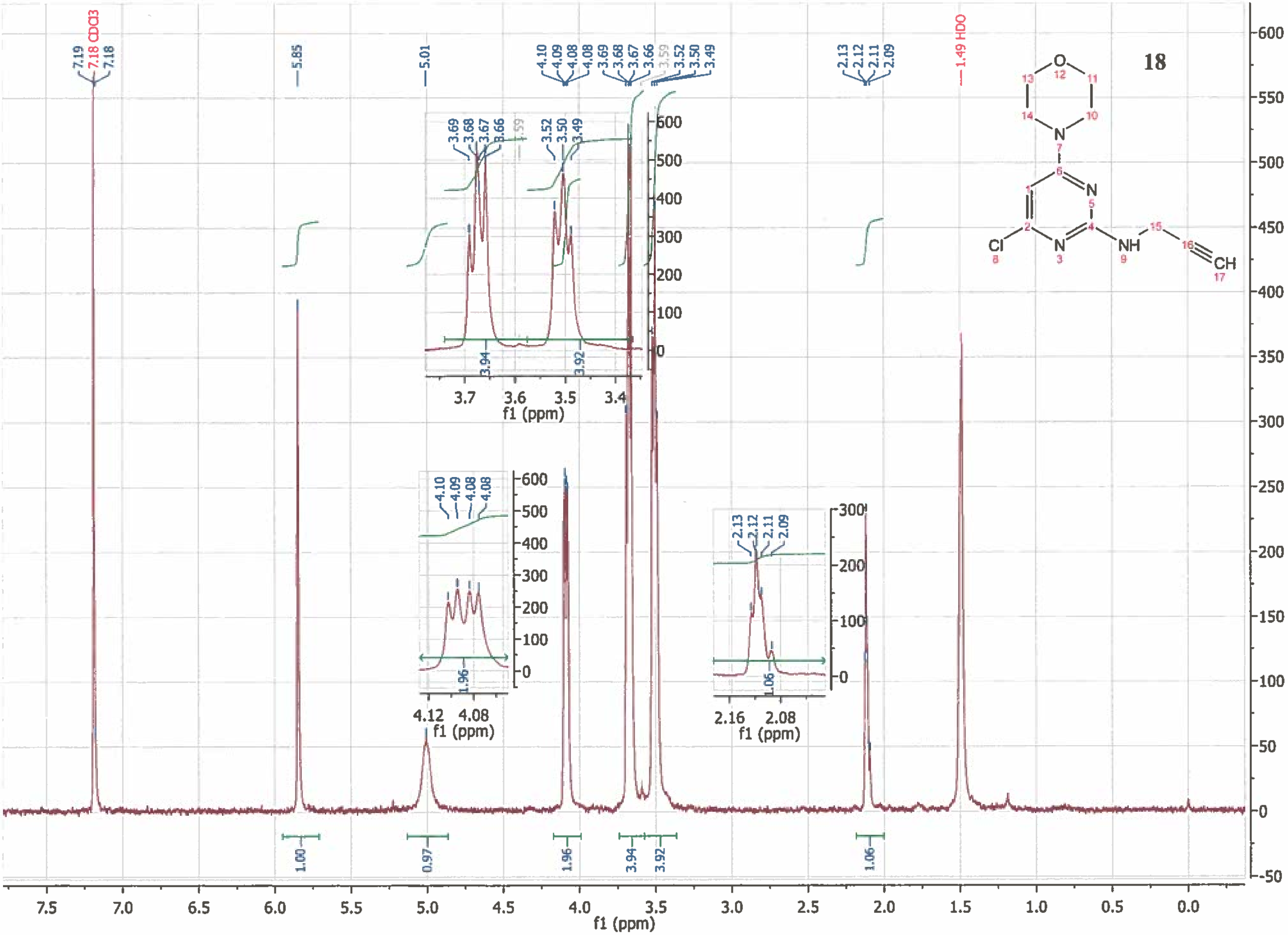
7.19
7.18 CDCl3
7.18
5.85
5.01
4.10
4.09
4.08
4.08
3.69
3.68
3.67
3.66
3.59
3.52
3.50
3.49
2.13
2.12
2.11
2.09
1.49 HDO
18
Cl
N
NH
CH
O
1.00
0.97
1.96
3.94
3.92
1.06
f1 (ppm)

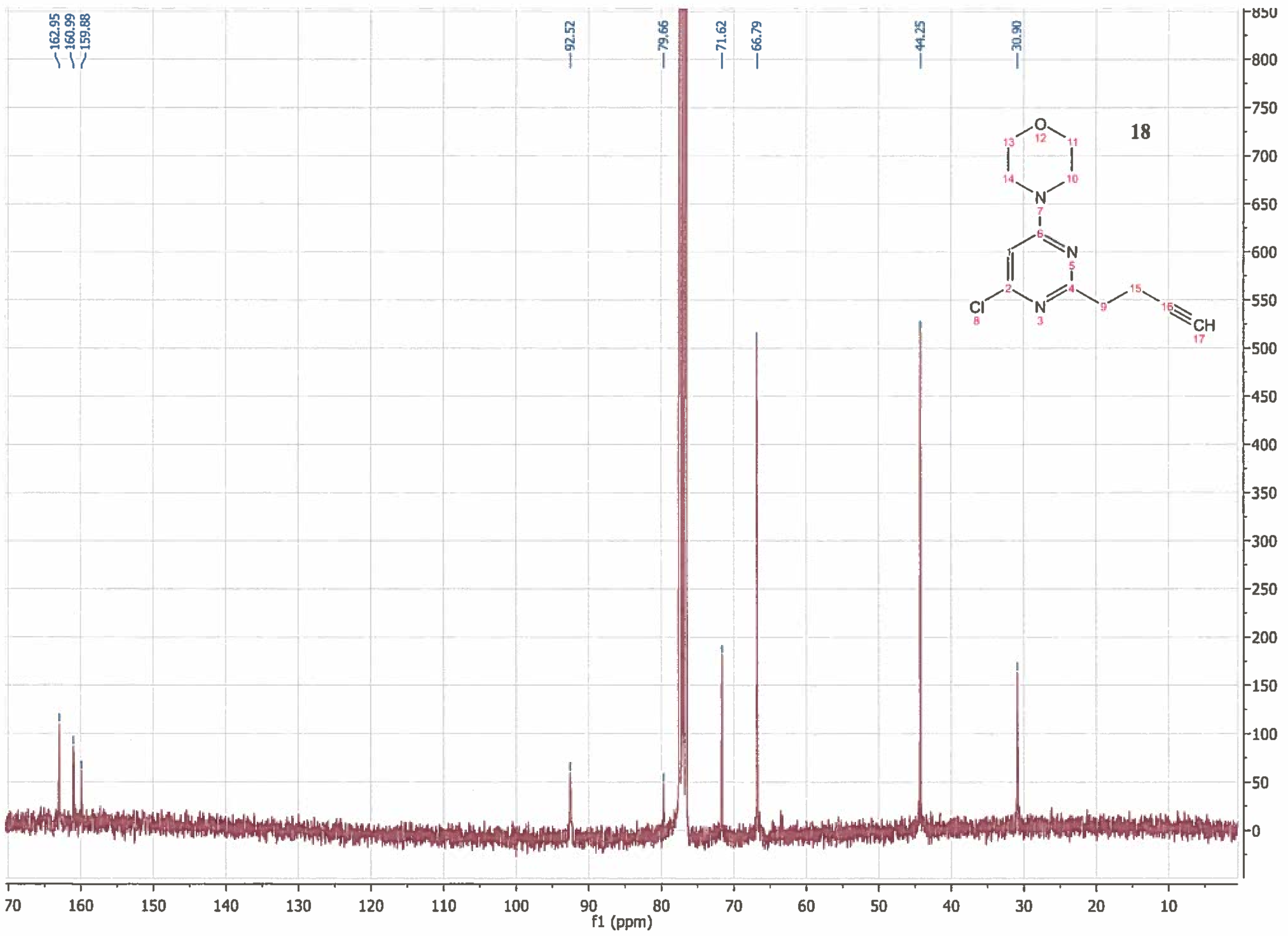
162.95
160.99
159.88
92.52
79.66
71.62
66.79
44.25
30.90
18
Cl
N
O
CH
f1 (ppm)

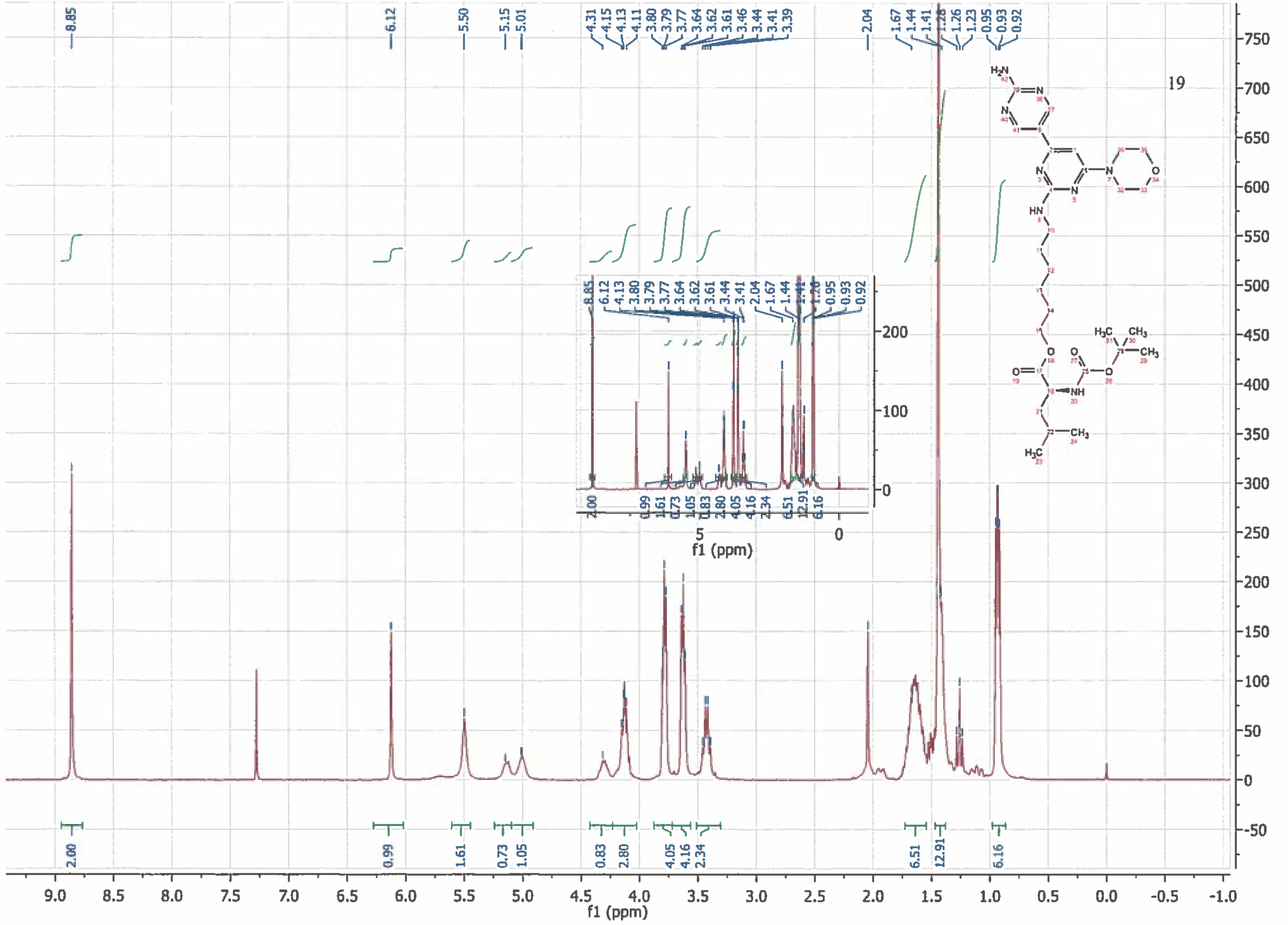

8.85
6.12
5.50
5.15
5.01
4.31
4.15
4.13
4.11
3.80
3.79
3.77
3.64
3.62
3.61
3.46
3.44
3.41
3.39
2.04
1.67
1.44
1.41
1.28
1.26
1.23
0.95
0.93
0.92
2.00
0.99
1.61
0.73
1.05
0.83
2.80
4.05
4.16
2.34
6.51
12.91
6.16
f1 (ppm)
H2N
HN
NH
O
N
H3C
CH3

19

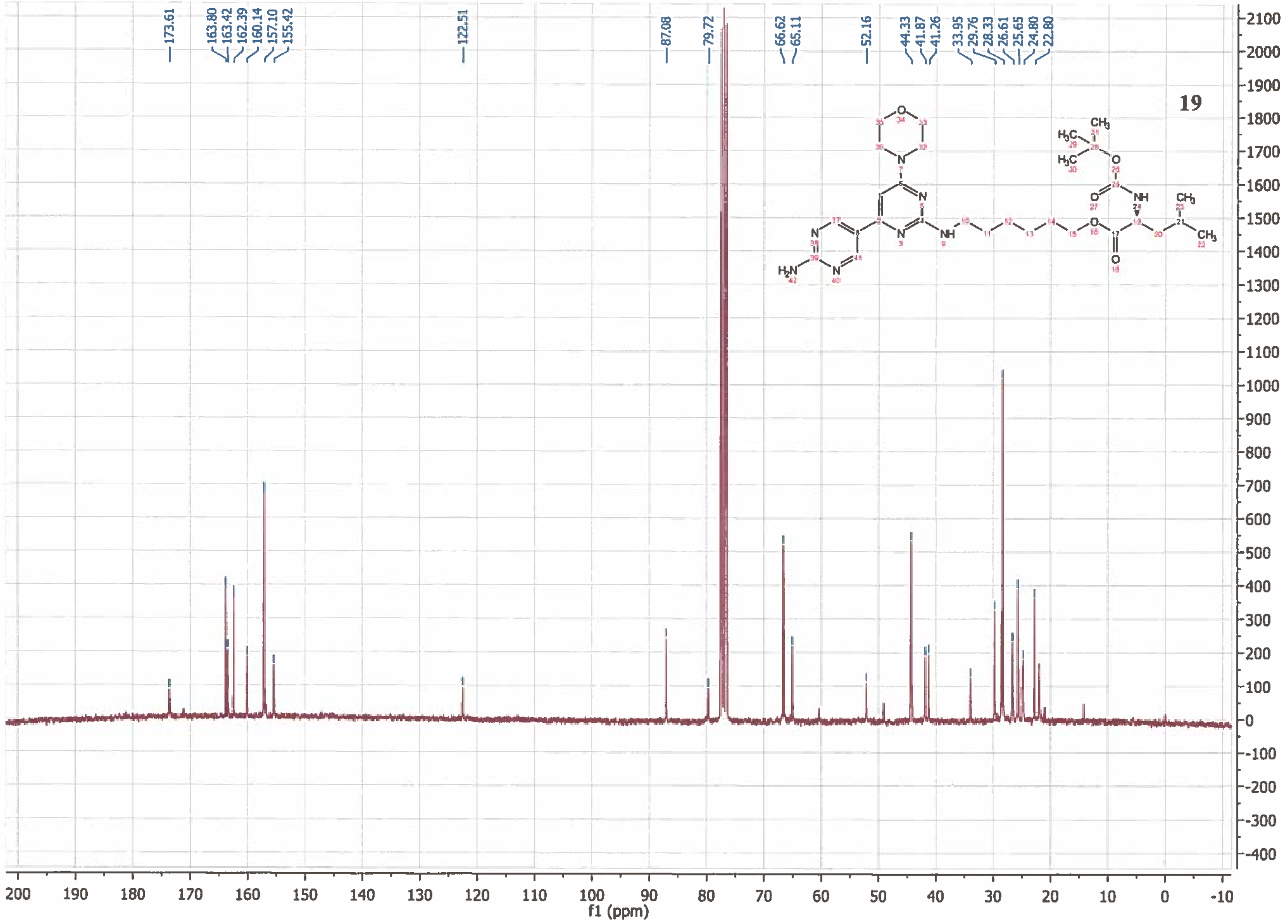
173.61
163.80
163.42
162.39
160.14
157.10
155.42
122.51
87.08
79.72
66.62
65.11
52.16
44.33
41.87
41.26
33.95
29.76
28.33
26.61
25.65
24.80
22.80
19
f1 (ppm)

20

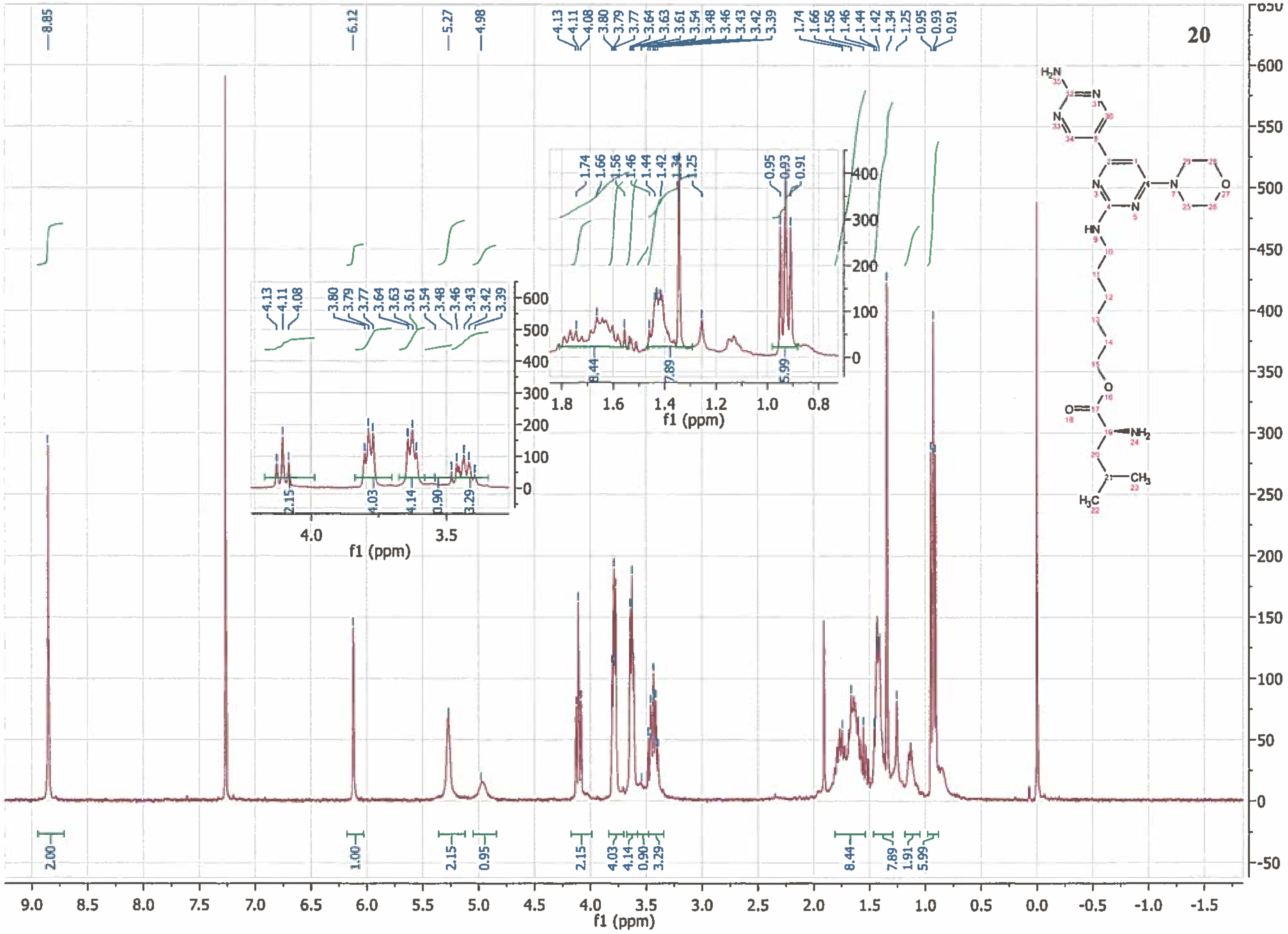

f1 (ppm)
8.85
6.12
5.27
4.98
4.13
4.11
4.08
3.80
3.79
3.77
3.64
3.63
3.61
3.54
3.48
3.46
3.43
3.42
3.39
1.74
1.66
1.56
1.46
1.44
1.42
1.34
1.25
0.95
0.93
0.91
2.00
1.00
2.15
0.95
2.15
4.03
4.14
0.90
3.29
8.44
7.89
1.91
5.99

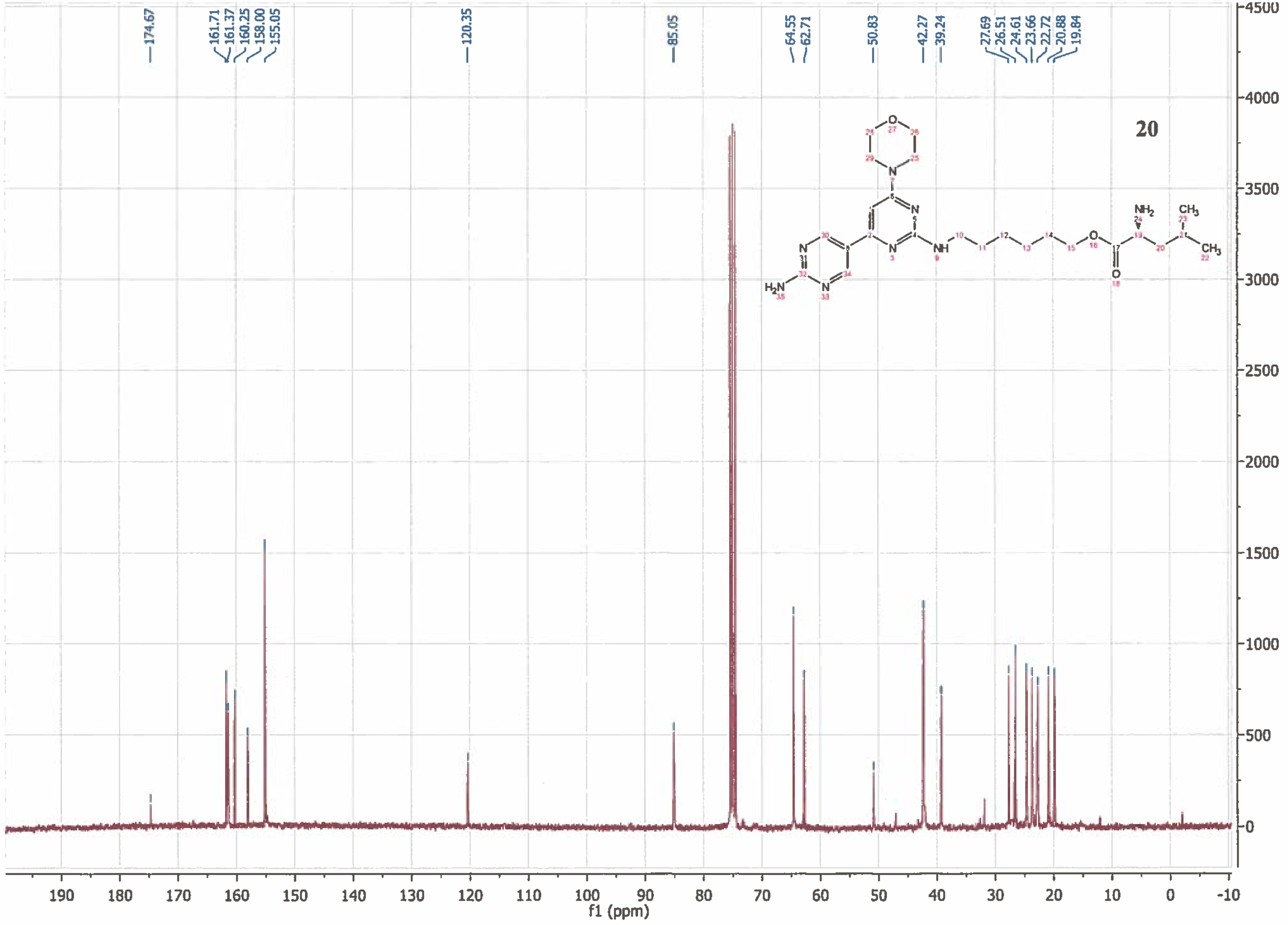

174.67
161.71
161.37
160.25
158.00
155.05
120.35
85.05
64.55
62.71
50.83
42.27
39.24
27.69
26.51
24.61
23.66
22.72
20.88
19.84
20
f1 (ppm)
